# Beneficial Outcome of Urethane Treatment Following Status Epilepticus in a Rat Organophosphorus Toxicity Model

**DOI:** 10.1523/ENEURO.0070-18.2018

**Published:** 2018-04-17

**Authors:** Asheebo Rojas, Wenyi Wang, Avery Glover, Zahra Manji, Yujiao Fu, Raymond Dingledine

**Affiliations:** Department of Pharmacology, Emory University, Atlanta, GA 30322

**Keywords:** DFP, diazepam, EEG, hippocampus, neurodegeneration, urethane

## Abstract

The efficacy of benzodiazepines to terminate electrographic status epilepticus (SE) declines the longer a patient is in SE. Therefore, alternative methods for ensuring complete block of SE and refractory SE are necessary. We compared the ability of diazepam and a subanesthetic dose of urethane to terminate prolonged SE and mitigate subsequent pathologies. Adult Sprague Dawley rats were injected with diisopropylfluorophosphate (DFP) to induce SE. Rats were administered diazepam (10 mg/kg, ip) or urethane (0.8 g/kg, s.c.) 1 h after DFP-induced SE and compared to rats that experienced uninterrupted SE. Large-amplitude and high-frequency spikes induced by DFP administration were quenched for at least 46 h in rats administered urethane 1 h after SE onset as demonstrated by cortical electroencephalography (EEG). By contrast, diazepam interrupted SE but seizures with high power in the 20- to 70-Hz band returned 6–10 h later. Urethane was more effective than diazepam at reducing hippocampal neurodegeneration, brain inflammation, gliosis and weight loss as measured on day 4 after SE. Furthermore, rats administered urethane displayed a 73% reduction in the incidence of spontaneous recurrent seizures after four to eight weeks and a 90% reduction in frequency of seizures in epileptic rats. By contrast, behavioral changes in the light/dark box, open field and a novel object recognition task were not improved by urethane. These findings indicate that in typical rodent SE models, it is the return of SE overnight, and not the initially intense 1–2 h of SE experience, that is largely responsible for neurodegeneration, accompanying inflammation, and the subsequent development of epilepsy.

## Visual Abstract

**Figure:**
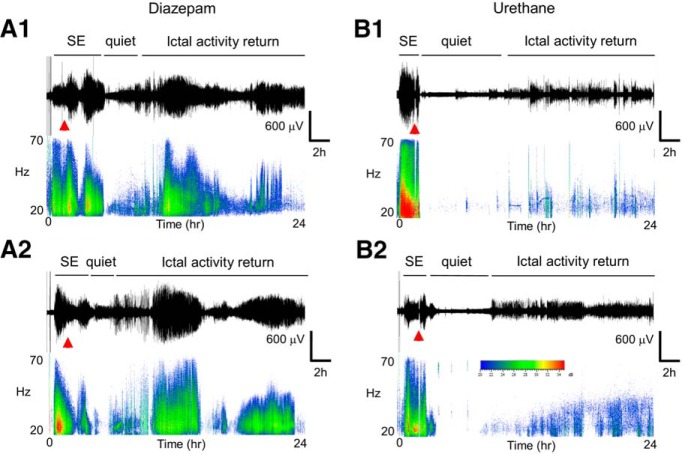

## Significance Statement

Delayed medical care to patients exposed to high levels of an organophosphorus (OP) agent can lead to the development of seizures and/or status epilepticus (SE). If left untreated, SE results in neuropathologies and the inevitable development of epilepsy in surviving rats. Administration of the general anesthetic, urethane following diisopropylfluorophosphate (DFP)-induced SE produced a number of beneficial consequences. Urethane effectively terminated electrographic SE, reduced neuroinflammation, neurodegeneration and astrogliosis, accelerated weight regain within 4 d after DFP exposure, and reduced the incidence and frequency of spontaneous recurrent seizures. This study gives insight into therapeutic modalities for the treatment of SE.

## Introduction

Status epilepticus (SE) is an unremitting seizure or a series of seizures without intervening regain of consciousness; in ∼30% of patients it is refractory to treatment with anti-epileptic drugs (AEDs) including benzodiazepines (Claassen et al., 2001; Mayer et al., 2002; Misra and Singh, 2002; Singhi et al., 2002; Holtkamp et al., 2005; Rossetti et al., 2005; Novy et al., 2010; Manno, 2011; Hunter and Young, 2012; Singh et al., 2014). Benzodiazepines like diazepam also become less effective as time in SE increases (Walton and Treiman, 1988, 1991; Kapur and Macdonald, 1997; Shih et al., 1999; Jones et al., 2002; Goodkin and Kapur, 2003; Goodkin et al., 2003; Löscher, 2007). In humans and rodents, prolonged exposure to high levels of an organophosphorus (OP) agent causes seizures, respiratory stress and cardiac failure if left untreated. The first line of treatment following OP poisoning includes administration of atropine to combat respiratory distress caused by increased bronchial secretions, and a benzodiazepine to terminate or reduce seizures and/or SE. Diazepam as a monotherapy is often ineffective in rodents at terminating SE that develops after OP poisoning, especially if SE persists for more than ∼40 min (McDonough et al., 2010; Todorovic et al., 2012; Reddy and Kuruba, 2013). However, diazepam is still currently approved by the US Food and Drug Administration as a first line treatment drug for OP nerve agent poisoning. Alternative methods for ensuring complete termination of SE are necessary.

Inhalational anesthetics such as isoflurane and desflurane are highly efficacious at terminating SE with a rapid onset of action and an ability to titrate the dose (Kofke et al., 1989; Mirsattari et al., 2004; Zhumadilov et al., 2014). However, these inhalational anesthetics often reduce blood pressure and increase heart rate in humans during anesthesia, compromising patient recovery (Tanaka et al., 1996; Mirsattari et al., 2004; Özarslan et al., 2012). The widely used veterinary anesthetic urethane (ethyl carbamate) can produce a subanesthetic state of drowsiness or a long-lasting deep level of anesthesia without affecting autonomic and cardiovascular systems (Soma, 1983; Maggi and Meli, 1986). Urethane reduces epileptiform activity and blocks the development of amygdala kindled seizures in rodents (Cain et al., 1989, 1992) as well as spinal seizures evoked by sudden cooling in a toad isolated spinal cord preparation (Piña-Crespo and Daló, 1992). The molecular mechanisms underpinning the anesthetic effects of urethane involve both inhibition of excitatory neurotransmitter receptors and potentiation of inhibitory transmitter receptors (Hara and Harris, 2002). Here, we tested the hypothesis that a subanesthetic dose of urethane aborts electrographic seizures following diisopropylfluorophosphate (DFP) exposure in adult rats and thus mitigates the ensuing pathology. The results shown here support a new strategy for the termination of prolonged SE and refractory SE following OP poisoning.

## Materials and Methods

### Ethics statement

All procedures concerning animals were approved by the Emory University School of Medicine’s Animal Care and Use Committee and conformed to the guidelines of the National Institutes of Health.

### Electrode implantation and electroencephalography (EEG) recording

Adult Sprague Dawley rats (240–420 g body weight) were implanted with monopolar electrodes on the surface of the cortex under general anesthesia [ketamine (100 mg/kg) and xylazine (10 mg/kg), i.p.]. The screw electrodes were made by attaching male miniature pin connectors (A-M Systems) to stainless steel screws with stainless steel wire (PlasticsOne). Two screw electrodes were positioned through burr holes above the left and right parietal cortices at the following coordinates (relative to bregma): AP, -3 mm and L, ±2 mm. A third surface electrode was positioned at the base of the frontal bone (AP, +4 mm and L, +2 mm; relative to bregma) and served as the reference. The screw electrodes penetrated the skull but not the dura matter and were secured to the skull with Instant Tray Mix (Lang Dental), which is a rapid self-curing dental acrylic resin that forms a hardened cap on the skull. Rats were then injected with yohimbine (8–9 mg/kg, s.c.) to reverse the effects of the anesthesia and meloxicam (1.5 mg/kg, s.c.) for pain management, and were allowed to recover from the surgery a minimum of 7 d before experimentation. On the day of EEG recording, wire leads with a female miniature pin (A-M Systems) were fastened to the screw electrodes on the skull of the rats. EEG signals were recorded using a Nicolet Endeavor CR system (VIASYS Healthcare) with a Tornado v32 amplifier (Natus Medical Inc) and the NicoletOne recorder software (VIASYS Healthcare). The EEG signals were bandpass filtered at 1–100 and 20–70 Hz and acquired at 500 Hz. We filtered the data at 20–70 Hz, as this γ-band frequency range minimizes noise and movement artifacts allowing for quantification of power that better represents the electrographic activity during SE (Lehmkuhle et al., 2009). Evaluation of acquired EEG was conducted by NicoletOne reader (VIASYS Healthcare), Spike2 version 8.09 (Cambridge Electronic Design) and programs written in Python 3.6.1. The EEG analysis software Spike2 was used to identify spikes and areas of high spiking, defined as abrupt and sharp transients that had an amplitude >2× of a 10-min baseline period before DFP administration; spikes and ictal activity were identified visually using the Spike2 sonogram feature. A script written in Python was used to obtain a running mean of EEG power in the 20- to 70-Hz band, defined as the square of the EEG amplitude signal, integrated over 300-s periods. An additional script in Python was created to remove large movement artifacts (single transients not of cerebral origin). EEG power was plotted as a function of time. The mean power during defined time periods after SE onset was obtained for each rat in the diazepam and urethane treatment groups.

### OP-induced SE

Adult Sprague Dawley rats (200–240 g body weight) were purchased from Charles River Labs and housed in standard plastic cages (two rats per cage) in a temperature-controlled room (22 ± 2°C) on a 12-h reverse light/dark cycle. Food and water were provided ad libitum. On the day of OP exposure, the rats were weighed, placed individually into a plastic cage and moved into a ventilation hood. Awake rats were injected subcutaneously with the reversible acetylcholinesterase inhibitor pyridostigmine bromide (P1339, TCI America) at 0.1 mg/kg in 0.9% saline. Twenty minutes later, the rats were injected subcutaneously with the muscarinic receptor antagonist methylatropine nitrate (A1596, Spectrum Chemicals) or ethylatropine bromide (Rojas et al., 2017) at 20 mg/kg in 0.9% saline. Pyridostigmine bromide and the muscarinic receptor antagonists, none of which appreciably crosses the blood-brain barrier, were administered to rats before DFP to reduce peripheral OP toxicity and increase survival of the rats following DFP exposure without altering the development of seizures. Ten minutes later, rats were injected with DFP (D0879, Sigma) diluted in sterile distilled water (9.5 mg/kg, i.p. or 5 mg/kg, s.c.). These two doses of DFP with the given route of administration result in a similar latency to SE onset and prolonged SE lasting >5 h without pharmacological intervention. Most of the rats (all diazepam and urethane administered) in the study were administered DFP at 5 mg/kg subcutaneously. Only a small cohort of normal adult male rats (from the uninterrupted group) were administered DFP at 9.5 mg/kg intraperitoneally. These rats were killed on day 4, and the neuropathology (neuroinflammation, neurodegeneration, and gliosis) was not different from rats that received DFP at 5 mg/kg subcutaneously (also in the uninterrupted group). DFP was prepared fresh within 5 min of administration with thorough mixing. Control (non-seizure) rats were treated similarly except they were given sterile water instead of DFP. Rats were administered diazepam (Hospira; 10 mg/kg, i.p.) 60 min after SE onset to interrupt SE. Additional subsets of rats were exposed for 5–7 min to isoflurane (Piramal Enterprises) by inhalation followed by administration of a subanesthetic dose of urethane (U2500, Sigma; 0.8 g/kg, s.c.) dissolved in physiologic saline 60 or 120 min after SE onset. Naïve rats injected with urethane (0.8 g/kg, s.c.) continuously displayed a strong hind leg flexion following a toe pinch as measured 5 min and 1 h following urethane administration. SE was not pharmacologically interrupted in an additional subset of rats (uninterrupted), whose seizures eventually waned. The uninterrupted rats were not administered a vehicle-control injection at the 60-min time point to ensure that SE was indeed uninterrupted. Each rat received a volume of injected compound based on weight (1 ml/kg).

### Behavioral scoring of seizure activity

In rats, OP-induced seizures begin within 10 min of DFP exposure and consist of distinct motor behaviors that include forelimb clonus, tail extension, and whole-body clonic seizures. Rats presenting these behaviors with increasing seizure intensity, duration, and frequency after exposure to DFP were declared to be in SE, which is characterized by nonintermittent whole-body clonic seizures that persist. A small subset of rats (∼15%) injected with DFP experienced occasional seizures but did not enter SE and were labeled “DFP no SE.” The seizure activity was scored and recorded every 5 min for 80–90 min using a modified Racine scale (Racine, 1972; see below). All rats were monitored for at least 6 h and persistent nonintermittent seizure activity was detected shortly after DFP exposure that lasted until pharmacological interruption 60 or 120 min post-SE onset, or until the seizures waned on their own. The rats were then placed individually into clean plastic cages with fresh bedding, soft food and water and allowed to recover. To maintain hydration, lactated Ringer’s solution (2 ml, i.p.) was administered when the rats were placed into cages and then daily until rats were able to eat and drink on their own. All rats were monitored and weighed daily.

### Modified Racine scale for DFP exposure

For modified Racine scale for DFP exposure, see Table 1.

**Table 1.** Modified Racine scale for DFP exposure

**Behavioral Score**		**Observed Motor Behavior**
0	Normal behavior	Walking, exploring, sniffing, grooming
1	Freeze behavior	Immobile, staring, heightened startle, curled-up posture
2	Repetitive behavior	Blinking, chewing, head bobbing, scratching, face washing, whisker twitching
3	Early seizure behavior	Myoclonic jerks, partial body clonus
4	Advance seizure behavior	Whole-body clonus
5	SE	Repeated seizure activity (≥2 events in stages 3, 4, or 6 within a 5-min window)
6	Intense seizure behavior	Repetitive jumping or bouncing, wild running, tonic seizures
7	Death	

### Modified Irwin test

A modified Irwin test (Irwin, 1968) was performed to access the health of rats before and after DFP-induced SE. The test is comprised of 12 parameters (ptosis, exophthalmos, lacrimation, body posture, bushy tail, tremors, running versus walking, dragging body, hyper-/hypoactive, aggression when handled, muscle tone when handled, and vocalization when handled) that can be measured simply by experimenter observation. Some parameters require agitating the rats by forcing them to move about their home cage. The test was given three times (once before DFP, 24 h after SE onset and finally on day 4 before being euthanized). Each parameter was scored on a three point scale (i.e., 0 = normal, 1 = mild to moderate impairment, and 2 = severe impairment) with a total score ranging from 0 to 24. A total score of 0 as the sum of all 12 parameters indicates a normal healthy rat. A total score ranging from 1 to 11 indicates a healthy animal that appears slightly impaired. A compromised animal would fail the modified Irwin test with a total score of ≥12.

### Quantification of CCL2 by ELISA

Protein lysates were generated from half brains (hemisphere without cerebellum) taken from DFP treated and non-seizure control rats on day 4 after DFP exposure, by homogenizing the tissue in RIPA lysis [25 mM Tris-HCl (pH 7.6), 150 mM NaCl, 1% Nonidet P-40, 1% sodium deoxycholate, and 0.1% SDS] and extraction buffer (Thermo Scientific) with proteinase and phosphatase inhibitors (Thermo Scientific). The lysates were incubated on ice for 2 h and then cleared by centrifugation at 14,000 × *g* for 15 min. The soluble fraction of the lysates was stored at −80°C until further use. The total protein level was measured by spectrophotometry using a SmartSpec 3000 (Bio-Rad) and using the Pierce protein assay (1861426, Thermo Scientific). The rat CCL2 ELISA was performed according to the manufacturer’s protocol provided with the kit (ELR-MCP1, RayBiotech).

### Acetylcholinesterase activity

The protein lysates described above were used to measure AChE activity using an acetylcholinesterase colorimetric assay kit (ab138871, Abcam) according to the manufacturer’s protocol. The assay uses 5,5’-dithio-bis(2-nitrobenzoic acid) (DTNB) to quantify thiocholine production by the hydrolyzis of acetylthiocholine by AChE in brain lysates. On the day of the assay the brain lysates were diluted and plated onto a 96-well plate (50 µl/well). Immediately after plating the acetylcholinesterase standards and unknowns, an acetylthiocholine reaction mixture (containing DTNB and acetylthiocholine; 50 µl/well) was added to the samples. Within 10 min the AChE activity was determined by measuring the change in the absorbance at 405 nm using a microplate reader. The data from the brain samples were normalized using the acetylcholinesterase standard curve according to the manufacturer’s protocol provided with the kit and compared to homogenates obtained from non-seizure control rats.

### FluoroJade B (FJB) histochemistry

Four days following DFP-induced SE a subset of rats was deeply anesthetized under isoflurane and decapitated. The brains were removed rapidly and longitudinally bisected. One hemisphere lacking the cerebellum of each brain was rapidly frozen on dry ice and kept for RNA isolation (below). The other half was fixed overnight in a 4% paraformaldehyde solution at 4°C and processed for immunohistochemistry (IHC) and FJB histochemistry. The half brains post-fixed in 4% paraformaldehyde were transferred to 30% (w/v) sucrose in PBS at 4°C the next day until they sank. The brains were embedded in tissue freezing medium (Electron Microscopy Sciences), slowly frozen, sectioned (40 μm) coronally through the hippocampus using a Cryostat (Leica CM 1850, Leica Biosystems). Every 12th hippocampal section from each rat brain was stained with FJB to label degenerating cells according to the manufacturer protocol (Histo-chem Inc.) as described by Schmued et al. (1997). Briefly, hippocampal sections were immersed in 100% ethyl alcohol for 3 min followed by a 1-min change in 70% alcohol and a 1-min change in distilled water. The sections were then transferred to a solution of 0.06% potassium permanganate and were gently shaken for 15 min on a rotating platform at 25 °C. The sections were rinsed for 1 min in distilled water and then transferred to 0.0004% FJB staining solution where they were gently agitated for 30 min. Following staining, the sections were rinsed with three 1-min changes of Tris-buffered saline (TBS), subsequently mounted onto clean slides and allowed to dry. The sections were made transparent with xylenes and then mounted under D.P.X. (Aldrich Chem. Co) mounting media. FJB labeling was visualized using an AxioObserver A1 epifluorescence microscope equipped with an AxioCam MRc 5 camera and a filter suitable for visualizing fluorescein or FITC (Zeiss). Pictures were taken using the software AxioVision AC 4.7 (Zeiss).

### Quantification of FJB-labeled cells

Following FJB labeling, images of three hippocampal areas (i.e., hilus, CA1, CA3) were taken with a 5× objective lens using the AxioVision AC 4.1 software (Zeiss) from each of 4 sections in each rat, from the dorsal hippocampus between bregma -2.56 and -4.16 mm (Paxinos and Watson, 1986). The number of bright FJB-positive cells in each hippocampal area was counted by an observer blinded to the treatment of the animals and the experimental conditions. The cell numbers were recorded and expressed as the mean and SEM number of positive FluoroJade B (+FJB) cells/section for each hippocampal area in each rat. FJB-stained sections were quantified for nine rats that received diazepam 1 h post-SE, seven rats that experienced uninterrupted SE, 15 rats that received urethane 1 h after SE, and eight rats that received urethane 2 h post-SE.

### IHC

Brains were prepared and sectioned as described above for FJB labeling. For IHC, the sections were separated for fluorescence and diaminobenzidine (DAB) immunostaining. For DAB staining, the sections were washed five times with TBS for 5 min each and then treated with 0.5% H_2_O_2_ for 30 min. The sections were washed to remove H_2_O_2_ and then blocked with TBS containing 1% bovine serum albumin (BSA), 10% goat serum, and 0.3% Triton X-100 at 25°C for 2 h, to reduce nonspecific immunostaining. The sections were then incubated in the primary antibodies [rabbit anti-Iba1 (1:2000), Wako; rabbit anti-GFAP (1:2000), Abcam] diluted in antibody dilution solution (ADS) containing 0.1% gelatin and 0.3% Triton X-100 in TBS at 4°C for 24 h. After washing three times with ADS, the sections were incubated with a goat anti-rabbit HRP-conjugated secondary antibody (Jackson ImmunoResearch) diluted to 1:500 in ADS for 4 h at 25°C. The sections were washed with TBS and exposed to DAB (DAB enhancement kit; Sigma-Aldrich). The sections were briefly exposed to a 0.1% cresyl violet solution for a Nissl counterstain. The floating sections were subsequently mounted onto clean slides and allowed to dry. After drying, the sections were dehydrated by exposure to increasing concentrations of ethanol, made transparent with xylenes and then cover slipped in the presence of permount.

For fluorescence IHC, the sections were washed three times with 1× PBS, blocked for 4 h in PBS containing 1% BSA, 10% goat serum, and 0.3% Triton X-100 at 25°C for 2 h. The sections were then incubated in the primary antibody [rabbit anti-cyclooxygenase-2 (COX-2) (1:1000), Abcam] diluted in ADS containing 0.1% gelatin and 0.3% Triton X-100 in PBS at 4°C for 24 h. After washing three times with ADS, the sections were incubated for 4 h at 25°C with Alexa Fluor goat anti-rabbit 488 (Invitrogen) diluted to 1:500 in ADS. The sections were washed three times with PBS and then incubated with the blue-fluorescent Hoechst 33342 dye (Invitrogen) diluted 1:2000 in ADS for 4 h at 25°C. The sections were again washed with PBS, mounted onto slides, allowed to dry, and cover slipped with FluoroGel mounting media. The DAB and fluorescence reactions were visualized using an AxioObserver A1 fluorescence microscope (Zeiss). Pictures were taken using the AxioVision AC 4.7 software (Zeiss). In control experiments, the sections were treated in a similar manner, except the primary antibodies were omitted. All negative control sections showed no staining (data not shown). All of the sections used for IHC were obtained from the dorsal hippocampus between bregma -2.56 and -4.16 mm (Paxinos and Watson, 1986).

### RNA isolation and quantitative real-time polymerase chain reaction (qRT-PCR)

Total RNA was isolated using Trizol with the PureLink RNA Mini kit (Invitrogen) from the frozen half brains lacking the cerebellum of rats that experienced SE and non-seizure controls. RNA concentration and purity were measured by a SmartSpec 3000 spectrophotometer (Bio-Rad) using the A260 value and the A260/A280 ratio, respectively. First-strand cDNA synthesis was performed with 1 μg of total RNA, using a qScript cDNA superMix kit (Quanta Biosciences) in a reaction volume of 20 μl at 25°C for 5 min and 42°C for 30 min. The reaction was terminated by heating at 85°C for 5 min. qRT-PCR was performed by using 8 μl of 50× diluted cDNA, 0.1–0.5 μM primers, and B-R iQ SYBR Green Supermix (Quanta Biosciences) with a final volume of 20 μl in the iQ5 Multicolor Real-Time PCR Detection System (Bio-Rad). Cycling conditions were as follows: 95°C for 2 min followed by 40 cycles of 95°C for 15 s and 60°C for 1 min. Melting curve analysis was used to verify specificity of the primers by single-species PCR product. Fluorescent data were acquired at the 60°C step. Real-time PCR primer sequences are listed in Table 2. The geometric mean of cycle thresholds for β-actin, glyceraldehyde-3-phosphate (GAPDH), and hypoxanthine phosphoribosyltransferase 1 (HPRT1; Table 3) was used as an internal control for relative quantification. Samples without cDNA template served as the negative controls. Of the 11 inflammatory mediators investigated (Table 2), three (IL-1β, IL-6 and TNFα) are produced and secreted by cells involved in both innate and acquired immunity to stimulate inflammation; four (CCL2, CCL3, CCL4, and CXCL10) are chemokines that recruit peripheral leukocytes to sites of inflammation or injury, although CXCL10 can also exert direct excitatory effects on neurons (Nelson and Gruol, 2004). COX-2 is an intracellular rate limiting enzyme in the production of prostanoids that contribute to the inflammatory response. Analysis of quantitative real time PCR data were performed by subtracting the geometric mean of the three internal control genes from the measured cycle threshold value obtained from the log phase of the amplification curve of each gene of interest. The fold change of each gene of interest was estimated for each animal 4 d after DFP-induced SE relative to the amount of RNA found in the control animals using the 2^-ΔΔ^*^C^* ^T^ method (Livak and Schmittgen, 2001). All conditions for qRT-PCR were the same.

**Table 2.** Real-time PCR primer sequences

**Genes**	**Forward Primer (Sequence 5’-3’)**	**Reverse Primer (Sequence 5’-3’)**
HPRT1	GGTCCATTCCTATGACTGTAGATTTT	CAATCAAGACGTTCTTTCCAGTT
β-ACTIN (ACTB)	CCAACCGTGAAAAGATGACC	ACCAGAGGCATACAGGGACA
GAPDH	GGTGAAGGTCGGTGTGAAC	CCTTGACTGTGCCGTTGAA
CCL2	CAGAAACCAGCCAACTCTCA	GTGGGGCATTAACTGCATCT
CCL3	TCCACGAAAATTCATTGCTG	AGATCTGCCGGTTTCTCTTG
CCL4	CATCGGAACTTTGTGATGGA	CACAGATTTGCCTGCCTTTT
CXCL10	GTGCTGCTGAGTCTGAGTGG	TTGCAGGAATGATTTCAAGTTTT
IL-1β (IL1B)	CAGGAAGGCAGTGTCACTCA	TCCCACGAGTCACAGAGGA
IL-6 (IL6)	AACTCCATCTGCCCTTCAGGAACA	AAGGCAGTGGCTGTCAACAACATC
TNFα (TNF)	CGTAGCCCACGTCGTAGC	GGTTGTCTTTGAGATCCATGC
COX-2 (PTGS2)	ACCAACGCTGCCACAACT	GGTTGGAACAGCAAGGATTT
BDNF	CGGAAACAGAACGAACAGAAAC	TGGCTCTCATACCCACTAAGA
TGFβ1 (TGFB1)	CTGGGCACCATCCATGAC	CAGTTCTTCTCTGTGGAGCTGA
GP91Phox (CYBB)	TGTGACAATGCCACCAGTCT	TCTTGCATCTGGGTCTCCA
GFAP	CATCTCCACCGTCTTTACCAC	AACCGCATCACCATTCCTG
lba1 (AIF1)	TCGATATCTCCATTGCCATTCAG	GATGGGATCAAACAAGCACTTC
CD11B (ITGAM)	GAGCATCAGTAGCCAGCAT	CCGTCCATTGTGAGATCCTT

The approved human gene nomenclature symbol is in parentheses if different from gene name

**Table 3.** CT values and geometric means of housekeeping genes in three groups of rats for 4-d inflammatory mediator measurement

Uninterrupted	β-Actin	GAPDH	HPRT1	Geomean
Control (5)	18.2 ± 0.1	17.3 ± 0.1	21.3 ± 0.1	18.9 ± 0.1
DFP-SE (7)	18.1 ± 0.1	17.3 ± 0.1	21.1 ± 0.1	18.8 ± 0.1
DFP no SE (4)	18.9 ± 0.3	17.7 ± 0.2	21.4 ± 0.2	19.2 ± 0.2
Diazepam-1 hr	β-Actin	GAPDH	HPRT1	Geomean
Control (7)	20.7 ± 0.3	19.4 ± 0.3	22.7 ± 0.2	20.9 ± 0.2
DFP-SE (9)	19.8 ± 0.4	19.1 ± 0.4	22.8 ± 0.3	20.5 ± 0.4
DFP no SE (4)	20.3 ± 0.3	19.3 ± 0.3	22.5 ± 0.2	20.6 ± 0.3
Urethane-1 hr	β-Actin	GAPDH	HPRT1	Geomean
Control (8)	21.0 ± 0.3	19.5 ± 0.2	22.6 ± 0.3	21.0 ± 0.2
DFP-SE (13)	19.7 ± 0.2	18.8 ± 0.2	21.9 ± 0.2	20.1 ± 0.2

The number in parentheses represents the number of rats in each group. Data are the mean ± SE.

### Light/dark exploration

Light/dark exploration testing was conducted to examine anxiety-related behavior in adult male and female rats that were administered DFP and non-seizure control rats. The test room was the same room the rats were housed in, but a different isolated cubicle to isolate odor, light intensity and noise. Light/dark preference was examined in all surviving rats four to five weeks following DFP exposure using a covered Plexiglas box (40 cm in length × 40 cm in width × 30 cm in height). The box is divided evenly by a partition that contained an opening (12 cm in height × 14 cm in width) located at the center of the floor into a light side with clear walls/cover and a dark side with black walls/cover as represented schematically in Figure 8*A*. On the test day each animal was placed in the dark compartment facing away from the opening and allowed to freely explore the entire apparatus for 5 min. Rat behavior and movement were recorded with a Sony handycam video camera (Sony) mounted directly above the apparatus and the videos were later analyzed by an observer blinded to the treatment groups. Each rat was tested only once. Time spent in the light, the number of entries into the lit compartment and latency to enter the light (with all four paws) were all used as measures of anxiety. The latency to the first head poke was defined as the elapsed time for the head of the rat to completely emerge through the opening between the light and dark compartments. The latency to the first head poke, the number of head pokes before the rat fully transitioned to the lit side, the number of full body crosses into the lit side and time spent in the lit side were all measured and compared among the three groups of rats. None of the rats experienced a generalized seizure in the light/dark box during testing.

### Novel object recognition (NOR) and open field tasks

A NOR test was used to assess recognition memory in adult male and female rats that had experienced SE four to five weeks earlier. The simple NOR testing paradigm used in the current study invokes minimal stress, which is an important consideration as the rats could develop seizures that may influence their performance during the task. Control rats along with rats that received a single injection of diazepam or urethane were tested. The NOR test was performed as previously described (Bevins and Besheer, 2006) with a lapse of 2 h between training and testing. NOR was conducted in a box similar to that used for light/dark exploration (40 × 40 × 30 cm). The test room was the same room the rats were housed in, but a different isolated cubicle to control for odor, light intensity and noise isolation. NOR testing consisted of three parts: habituation, training/object familiarization and NOR testing (Fig. 9*A*). Several pairs of appropriate objects were used for familiarization and testing. Adult Sprague Dawley rats that had experienced SE four to five weeks earlier and controls were transported individually to the testing arena in their home cages for the three parts of the test. During the habituation session rats were placed in the empty arena and allowed to freely move about, and their distance traveled was analyzed. Time in the center was measured during the pretraining phase. On the next day habituation was repeated for 5 min. Following the habituation, the rats were briefly removed from the arena for ∼5 min and then returned for object familiarization that was conducted once, however now the arena contained two identical objects 20 cm apart and total time spent exploring the identical objects was recorded for 5 min. Novel object testing was performed 2 h after object familiarization in the same manner except one of the objects was replaced by a novel object. The rats were allowed to freely move about the box and explore the objects for 5 min. Object exploration was later scored in its entirety from video recordings of the trial by an experimenter blinded to the treatment of the rats during testing. Object exploration was defined as orientation of the head toward the object with the nose within 1 cm of the object with behaviors including sniffing, touching and gnawing. Excluded from the total exploration time was any time spent with the object where a rat simply propped the forepaws onto the object with the nose pointing away from the object. Rats were evaluated for their ability to remember the familiar object by expressing a preference for exploring the novel object. Preference for the novel object was expressed as a discrimination index (DI; equation below), which compares the amount of time a rat spent exploring the novel object compared to the familiar object. Which object served as the novel object and the left/right position of the objects were changed between rats within each group. NOR scoring was later analyzed visually by an observer blinded to the treatment groups. Between each trial, the arena and the objects were cleaned with 30% isopropyl alcohol and then dried with paper towels to remove any trace odors.

$$DI=\frac{\left(time\,\;exploring\,\;novel\,\;object-time\,\;exploring\,\;familiar\,\;object\right)}{\left(time\,\;exploring\,\;novel\,\;object+time\,\;exploring\,\;familiar\,\;object\right)}$$

### Spontaneous recurrent seizure recording

Normal adult male and female rats were administered DFP as described above to induce SE. Two weeks following DFP exposure EEG electrode implantation surgery was conducted as described above. Two weeks after the surgery the rats were connected to a Nicolet Endeavor CR system (VIASYS Healthcare) with a Tornado v32 amplifier (Natus Medical Inc) and the NicoletOne recorder software (VIASYS Healthcare). EEG signals were recorded continuously 24 h/d from freely moving rats for a total of 6–12 d and stored on a computer disk for later analysis. Evaluation of acquired EEG was conducted by NicoletOne reader, Spike2 software and Python 3.6.1 scripts. Spike2 was used to identify spikes and areas of high spiking, defined as abrupt and sharp transients that had an amplitude >3× of a 5-min baseline period; spikes and ictal activity were identified visually using the sonogram feature in Spike2. A script written in Python was used to obtain EEG power defined above. Spontaneous recurrent seizures were identified visually in Spike2 based on the criteria below by an observer that was blinded to the treatment of the rats. A second observer blinded to the treatment of the rats visually identified SRS based on the same criteria. The number of seizures detected by both observers were compared and found to be identical. The total number of seizures was determined for each rat. The period of high-intensity spiking during an ictal event was measured in Spike2 and presented as the seizure duration.

### Spontaneous recurrent seizure detection criteria

1. Normal baseline activity for a minimum of 5 min prior to an event
2. Gradual (not abrupt) ramp up of spiking, typically over several seconds
3. Continuous high-frequency spiking (3× greater amplitude than baseline) that lasts >20 s
4. Abrupt shutoff of spiking (noticeable stop to high-frequency spiking)
5. Clear post-ictal suppression that lasts ≥30 s (even if there is after discharge)

### Data analysis

Data are presented as mean ± SEM. Statistical analysis was performed with GraphPad Prism version 4 or 5 (GraphPad software). Student’s *t* test, Mann–Whitney test, Kruskal–Wallis test (with Dunn’s *post hoc*), or one-way ANOVA (with Bonferroni *post hoc* tests) was performed as appropriate to examine differences of chemical effects. Fisher’s exact test was used to compare mortality rates, the percentage of rats that entered SE, the percentage of rats that displayed >10 +FJB cells in hippocampal subregions and the percentage of rats that experienced at least one spontaneous recurrent seizure. The differences were considered to be statistically significant if *p* < 0.05. The Shapiro–Wilk test in Origin 9.4.2 (OriginLab) was used to test normality of the data. Experimental power analysis was performed with GPower3.1 (Universitat Kiel, Germany) and R (using a Monte Carlo simulation). The observed power values are presented in Table 4.

**Table 4.** Statistical table

**Data Structure**	**Type of Test**	**Observed Power**	***p* Value**
a Normally distributed	Unpaired *t* test	0.05	0.82
b Normally distributed	Unpaired *t* test	0.90	0.004
c Categorical	Fisher’s exact test	0.05	0.60
d Normally distributed	One-way ANOVA with *post hoc* Bonferroni	0.99	>0.05
e Normally distributed	two-way ANOVA with *post hoc* Bonferroni	0.61 (t), 0.99 (d), 0.49 (i)	<0.05 (d3), <0.01 (d4)
f Normally distributed	One-way ANOVA with *post hoc* Bonferroni	0.81	>0.05 (d4)
g Categorical	Fisher’s exact test	0.05	0.60
h Normally distributed	One-way ANOVA with *post hoc* Bonferroni	0.38	>0.05
i Log-normal distribution	Mann–Whitney test	0.99	0.0001
j Log-normal distribution	Mann–Whitney test	0.83	0.0003
k Categorical	Fisher’s exact test	0.98	0.0005
l Categorical	Fisher’s exact test	0.96	0.0007
m Normally distributed	Unpaired *t* test	0.05	0.99
n Categorical	Fisher’s exact test	0.59 (CA1), 0.53 (CA3)	0.050 (CA1), 0.057 (CA3)
o Normally distributed	Paired *t* test	0.83	0.008
p Normally distributed	One-way ANOVA with *post hoc* Bonferroni	0.77	<0.001
q Normally distributed	One-way ANOVA with *post hoc* Bonferroni	0.82	<0.001
r Normally distributed	One-way ANOVA with *post hoc* Bonferroni	0.82	>0.05
s Skewed distribution	Kruskal–Wallis test with *post hoc* Dunn’s	0.99	<0.001 (dzp), <0.01 (urethane)
t Skewed distribution	Kruskal–Wallis test with *post hoc* Dunn’s	0.84	>0.05
u Normally distributed	One-way ANOVA with *post hoc* Bonferroni	0.05	>0.05
v Categorical	Fisher’s exact test	0.93	0.001
w Normally distributed	Unpaired *t* test	0.55	0.01

Power: t = treatment, d = days, I = interaction; *p* value: d3 = day 3 and d4 = day 4; dzp = diazepam.

## Results

**Figure 1.**
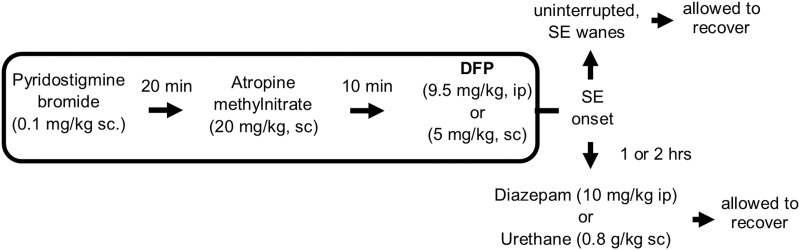
Experimental paradigm of chemical administration in a rat model of DFP-induced SE. All rats were administered pyridostigmine bromide and atropine methylnitrate or ethylatropine bromide (Rojas et al., 2017) followed by DFP to induce SE. For the diazepam cohort, rats were injected with one dose of diazepam 1 h after DFP-induced SE onset. For urethane treatment following DFP, rats were injected with a single dose of urethane 60 or 120 min from the onset of SE. A separate cohort of rats was not administered any drugs after SE onset (uninterrupted SE). All rats were allowed to recover and were monitored for 4 d.

DFP produces SE in adult Sprague Dawley rats that lasts at least 5 h without pharmacological intervention (Rojas et al., 2015). Rats were either administered diazepam (10 mg/kg, i.p.) or urethane (0.8 g/kg, s.c.) to interrupt SE 1 or 2 h after SE onset. In a separate cohort of rats SE was uninterrupted pharmacologically and waned on its own after several hours (Fig. 1).

### Urethane dampens the return of seizure activity following DFP-induced SE

**Figure 2.**
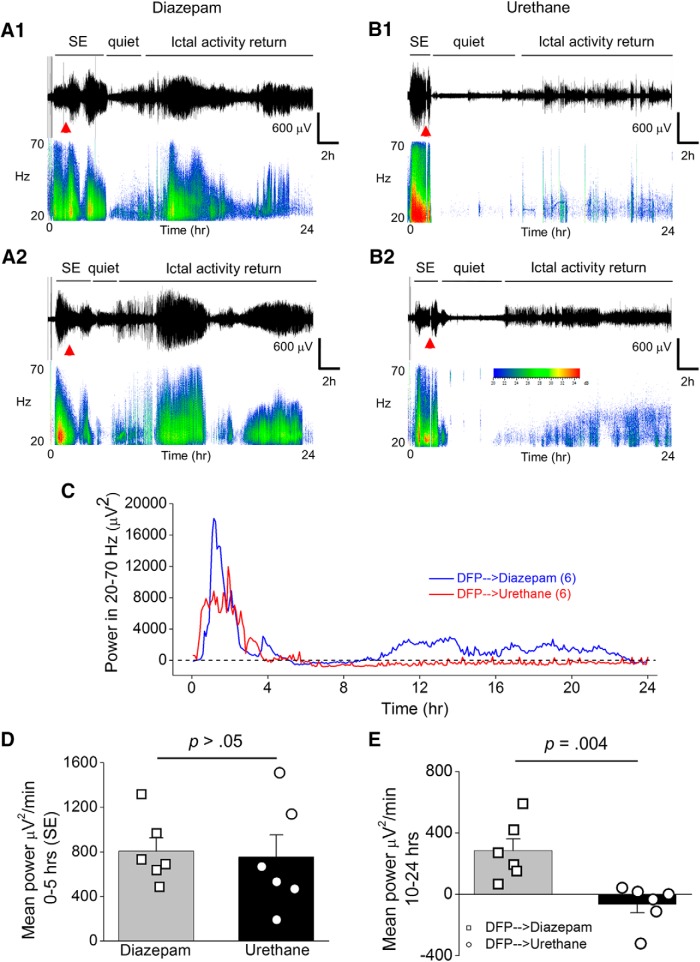
Urethane, but not diazepam, suppresses the return of seizure activity following 1 h of DFP-induced SE. ***A1***, Cortical EEG activity was recorded prior and during SE induced by exposure to DFP for 24 h. A representative EEG trace and sonogram from the cortical recording of an adult male rat showing increased spike activity just after exposure to DFP that develops into SE (denoted by the bar above the trace). Spike activity was quieted by administration of diazepam (10 mg/kg, i.p.) 1 h after SE onset (red triangle) but returned within a few hours as denoted by the ictal activity return phase. ***A2***, is a similar EEG trace from the cortical recording of a second adult male rat that received diazepam 1 h after SE onset. ***B1***, A representative EEG trace from the cortical recording of an adult male rat shows increased spike activity just after exposure to DFP that developed into SE (denoted by the bar above the trace), which was quieted following brief exposure to isoflurane (inhaled) and subsequent injection of urethane (0.8 g/kg, s.c., red triangle) 1 h after SE onset. ***B2***, Similar EEG trace from the cortical recording of a second adult male rat that received isoflurane and subsequent injection of urethane 1 h after SE onset. Below each raw EEG trace is a sonogram of the spike activity obtained in Spike2. The colors of the sonogram indicate the spectral power density in decibels (dB) at the indicated frequency. ***C***, the EEG power in the 20- to 70**-**Hz bandwidth averaged over 300**-**s epochs during the 24**-**h period for six diazepam-treated and six urethane-treated rats. The dashed line indicates baseline power before DFP. ***D***, During the first 5 h after DFP administration, no difference was detected between the two treatment groups (*n* = 6 for both diazepam and urethane) in the EEG power in the 20- to 70**-**Hz bandwidth. Error bars show SEM; *p* > 0.05, Student’s *t* test. ***E***, A difference was detected between the two treatment groups [diazepam (*n* = 6) and urethane (*n* = 6)] in the EEG power in the 20- to 70**-**Hz bandwidth using power analysis during the period 10–24 h after SE. Error bars show SEM; *p* = 0.004, Student’s *t* test. The symbols in ***D***, ***E*** represent each individual rat within the group.

**Figure 3.**
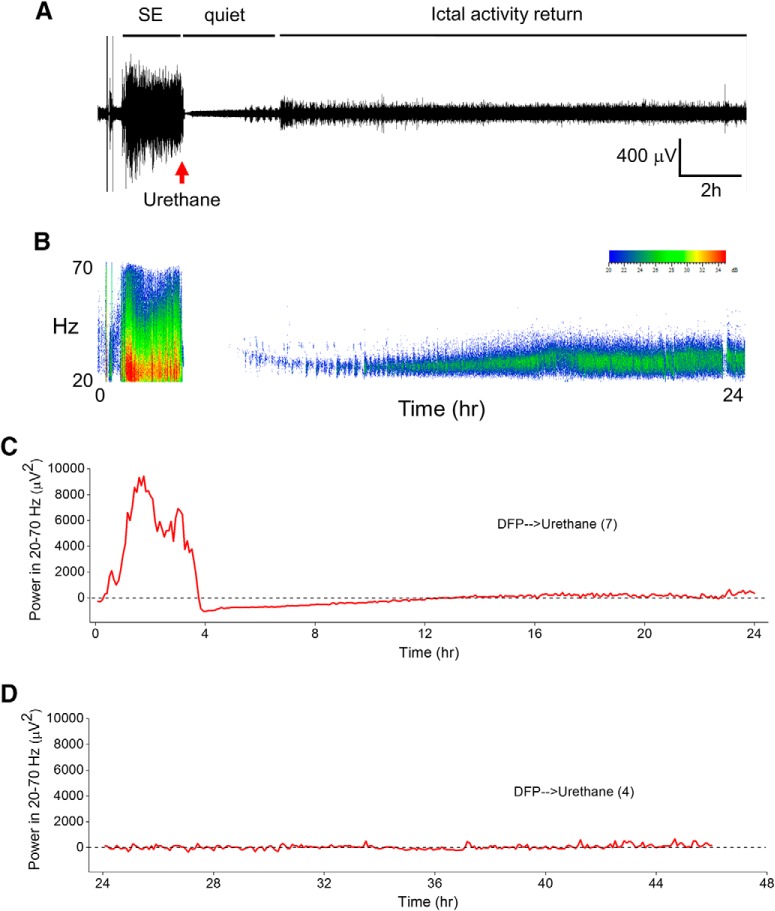
Administration of urethane 2 h after DFP-induced SE onset terminated SE and suppressed the return of seizure activity. ***A***, Cortical EEG activity was recorded prior and during SE induced by exposure to DFP for 24 h. A representative EEG trace from the cortical recording of an adult male rat showing increased spike activity just after exposure to DFP that developed into SE (denoted by the bar above the trace). Spike activity was quieted by brief exposure to isoflurane (gas, inhaled) and subsequent injection of urethane (0.8 g/kg, s.c.) 2 h after SE onset but returned within a few hours as denoted by the ictal activity return phase. ***B***, Sonogram of the spike activity obtained in Spike2 for the 20- to 70**-**Hz bandwidth. ***C***, Diagram showing the total EEG power in the 20- to 70**-**Hz bandwidth using power analysis during the first 24 h and the next day (***D***). The dashed line indicates no spiking activity.

To determine the efficacy of diazepam and urethane to terminate SE we performed cortical EEG before, during DFP exposure and after administration of the anesthetics. Adult male rats were instrumented with bilateral cortical electrodes 7–10 d before EEG recording and DFP exposure. On the day of experiment rats were connected to the EEG instrument and their baseline brain activity was recorded for at least 20 min before DFP. All rats tested displayed normal cortical activity before any drug administration as determined by the low amplitude, frequency and shape of the waveforms (Fig. 2*A*,*B*). EEG was recorded continuously for 24–46 h in all rats. Exposure to DFP led to the appearance of seizures defined by the sudden onset of a burst of large-amplitude (>2× the baseline before drug treatment) and high-frequency spikes. The onset of SE was defined as the appearance of the initial electrographic seizure that consisted of large-amplitude spikes that persisted for >10 s followed by a rapid quieting of electrical activity. Recurring large-amplitude, high-frequency spikes persisted following the first seizure (Fig. 2*A*,*B*). All rats received a single injection of DFP and the latency to SE onset was very similar. Both diazepam and urethane reduced the large-amplitude high-frequency spikes and thus interrupted SE (Fig. 2*A*,*B*). A script written in Python was used to obtain EEG power in the 20- to 70-Hz band in 300-s epochs over the 24-h EEG recording. Using the EEG power analysis, we determined mean power for time 0–5 h in every recording as a measure of the intensity of SE. During SE the mean power for the first 5 h was not different for rats administered diazepam (805 ± 121 μV^2^/min, *n* = 6 rats) after SE compared to rats administered urethane (753 ± 198 μV^2^/min, *n* = 6 rats; *p* > 0.05, Student’s *t* test^a^; Fig. 2*C*,*D*). However, ictal activity returned in all rats and the mean power measured between 10–24 h was significantly greater for rats administered diazepam 1 h after SE onset (284 ± 79 μV^2^/min, *n* = 6 rats) compared to rats given urethane (-66 ± 55 μV^2^/min, *n* = 6 rats; *p* = 0.004, Student’s *t* test^b^; Fig. 2*E*). Urethane was also effective at blocking the return of large-amplitude high-frequency spiking following 2 h of SE with a mean power of 650 ± 171 μV^2^/min (*n* = 7) for 0–5 h and 15 ± 38 μV^2^/min (*n* = 7 rats) for 10–24 h (Fig. 3). It should be noted that two rats displayed small amplitude spikes during SE in the 2 h SE group thus reducing the mean power. All urethane rats displayed low power spiking activity throughout the ictal activity return phase following urethane administration although spiking with high power in the 20- to 70-Hz band did not recur even when recordings were continued up to 48 h (Fig. 3). Before the urethane injection, rats were exposed briefly to isoflurane to induce a quick effect and mimic the rapid behavioral sedative effect of diazepam. Brief exposure to isoflurane alone was not effective in terminating SE as high-frequency and large-amplitude spiking indicative of SE returned within 5 min after the effect of isoflurane wore off (data not shown). Together, these data demonstrate that urethane is more effective than diazepam at terminating DFP-induced SE that lasts for at least 1 h, and in contrast to diazepam, urethane reduces the subsequent overnight return of high-power seizure activity.

### Urethane improves functional recovery after DFP-induced SE

**Figure 4.**
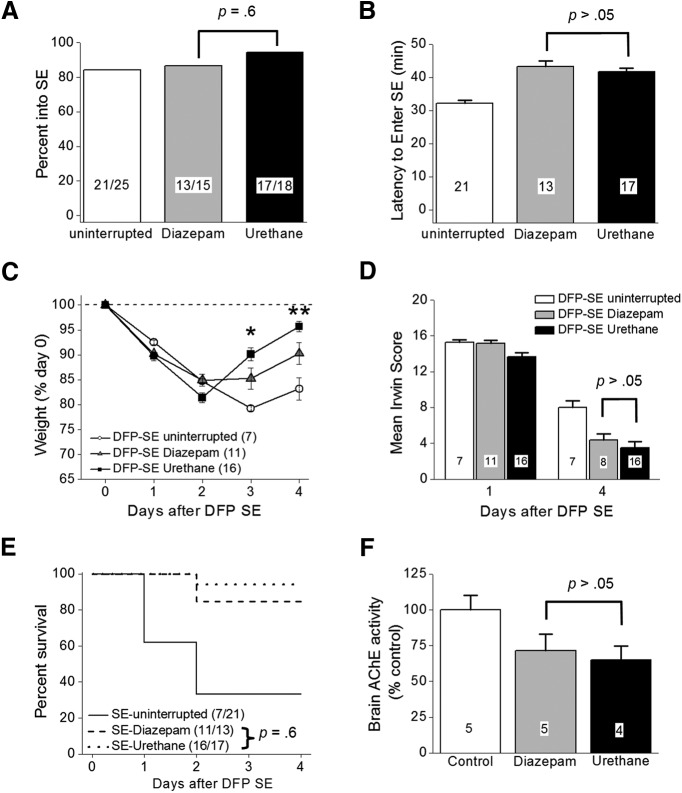
DFP-induced SE characteristics. ***A***, Percentage of rats entering SE following a single intraperitoneal injection of DFP was not different between the groups (uninterrupted SE, diazepam-treated rats and urethane-treated rats; *p* = 0.6, Fisher’s exact test) of rats administered diazepam compared to urethane-injected rats 1 h after SE. ***B***, Latency to the onset of SE following a single intraperitoneal injection of DFP in different groups of rats. (*p* > 0.05, one-way ANOVA with *post hoc* Bonferroni). The number inside the bar represents the number of rats from each group. ***C***, Single injection of urethane (*n* = 16 rats) 60 min after SE onset significantly accelerated weight regain compared to 11 rats treated with diazepam (*p* < 0.05 day 3 and *p* < 0.01 day 4 by two-way ANOVA with *post hoc* Bonferroni). Rats administered DFP that did not receive anything after SE (*n* = 7) minimally began gaining weight on day 4. The dashed line indicated the original weight of the rats before any manipulation on day 0. ***D***, There was no difference in the mean Irwin score measured 24 and 4 d after SE of 11 rats administered diazepam following DFP (gray bars) compared to 16 rats administered urethane (black bars; *p* > 0.05, one-way ANOVA with *post hoc* Bonferroni). In the diazepam group (gray bars), we killed three rats without obtaining the day 4 Irwin score (simply forgot to score them) and this attributed to the difference in the number of rats on day 1 compared to day 4. ***E***, Survival rates of 11 rats that received diazepam, urethane (*n* = 16 rats), or nothing (*n* = 7 rats) up to day 4 after DFP-induced SE. No difference was detected in the survival rate for rats administered diazepam (11 of 13 rats survived) compared to urethane (16 of 17 rats survived) on days 1–4 after DFP-induced SE (*p* = 0.6, Fisher’s exact test). ***F***, Inhibition of acetylcholinesterase in rat forebrain on day 4 after DFP exposure; *p* > 0.05, one-way ANOVA with *post hoc* Bonferroni. The number inside the bar represent the number of rats in each group. The controls were non-seizure rats that were administered pyridostigmine bromide, methylatropine nitrate, and water instead of DFP. Shown in ***B***, ***D***, ***F*** are the mean ± SEM.

Experiments were performed with non-EEG instrumented rats to investigate the importance of terminating DFP-induced SE and the consequential functional recovery during the first 4 d. DFP was administered to 84 adult male Sprague Dawley rats, of which 70 entered SE resulting in an 83% success rate. The remaining 14 rats displayed episodic seizure like activity but failed to develop nonintermittent seizure activity, and were given the label DFP no SE. The percentage of rats that entered SE was not different regardless of whether they received diazepam (13 out of 15 rats, 87%) or urethane (17 out of 18 rats, 94%) following 1 h of DFP-induced SE (Fig. 4*A*; *p* = 0.6, Fisher’s exact test^c^), and similar to that of rats not administered drugs following DFP-induced SE (21 out of 25 rats, 84%). The latency to enter SE following DFP exposure was similar for rats injected with either diazepam (43 ± 2 min, *n* = 13 rats) or urethane (42 ± 1 min, *n* = 17 rats) 1 h after SE onset (Fig. 4*B*; *p* > 0.05, one-way ANOVA with *post hoc* Bonferroni^d^). Together with Figures 2*D*, 4F, these data indicate that rats administered diazepam or urethane experienced similar intensities of SE.

Rats that experience DFP-induced SE usually lose body weight for the first 2 d and then may start to regain weight the days following (Rojas et al., 2015, 2016). All rats exposed to DFP lost a similar amount of weight on day 1 (∼9% average; Fig. 4*C*). However, by day 3, rats that received diazepam 1 h after SE onset stabilized their weight and by day 4 they gained weight (Fig. 4*C*, gray triangles). DFP-injected rats that received urethane 1 h after SE began to regain weight by day 3 and by day 4 returned close to their original weight (before DFP; Fig. 4*C*, black squares). The weight change on day 4 was significantly different for urethane administered rats compared to diazepam injected rats (96 ± 1% of day 0 for urethane, *n* = 16 rats; 90 ± 2% of day 0 for diazepam, *n* = 11 rats; *p* < 0.01, two-way ANOVA with *post hoc* Bonferroni^e^; Fig. 4*C*). By contrast, rats experiencing SE without interruption continued to lose weight on day 3 and only modestly gained weight on day 4 (Fig. 4*C*, open circles).

Using a modified Irwin test, we determined the effect of diazepam and urethane on a selective subset of normal physiologic characteristics of rats before treatment on day 0, 24 h, and again 96 h after DFP-induced SE. All rats subjected to the modified Irwin test before treatment on day 0 scored 0 (data not shown) indicative of normal rat health and behavior. However, all rats that experienced SE regardless of post-SE treatment failed (score ≥12) the modified Irwin test measured 24 h after DFP induced SE (Fig. 4*D*), consistent with compromised health and the 24-h weight loss in Figure 4*C*. None of the rats that failed the Irwin test showed signs of infection from the multiple injections on day 0, but instead they displayed lethargy. The rats that were administered urethane remained sedated at the 24-h time point but were clearly not anesthetized as they displayed reflex responses to agitation. The mean 24-h modified Irwin test score for rats that experienced uninterrupted SE was 15.3 ± 0.3 (*n* = 7 rats; Fig. 4*D*). Similarly, rats that received diazepam or urethane displayed compromised health determined 24 h after SE onset SE (15.2 ± 0.3 for diazepam, *n* = 11 rats; 13.6 ± 0.5 for urethane, *n* = 16 rats). By day 4, all surviving rats displayed a reduced modified Irwin test score. However, no difference was detected in the 4 d modified Irwin test score of rats that were administered diazepam or urethane after SE (4.4 ± 0.7 for diazepam, *n* = 8 rats; 3.5 ± 0.7 for urethane, *n* = 16 rats; *p* > 0.05, one-way ANOVA with *post hoc* Bonferroni^f^; Fig. 4*D*), suggesting that termination of SE that persists for at least 60 min does not alter the 12 characteristics measured at 24 h after SE. This is consistent with the lack of effect of urethane on weight loss measured at 1 d after SE (Fig. 4*C*,*D*).

In contrast to the low percentage survival observed in the current study in rats that endured long lasting SE without pharmacotherapy (seven of 21 rats), rats that survived the acute episode of SE elicited by DFP administered diazepam or urethane usually survived the next 4 d (Fig. 4*E*). For example, 11 of 13 rats administered diazepam 1 h after SE onset survived over the 4-d period, whereas 16 of 17 rats administered urethane survived (*p* = 0.6, Fisher’s exact test^g^). Brain acetylcholinesterase activity was measured on day 4 in rats administered water instead of DFP (non-seizure controls) and compared to the acetylcholinesterase activity of DFP-SE rats administered diazepam or urethane 1 h after SE onset. No significant difference was detected in acetylcholinesterase activity in diazepam-treated rats versus urethane-injected rats (71.5 ± 11% of control for diazepam, *n* = 5 rats; 65.1 ± 10% of control for urethane, *n* = 4 rats; *p* > 0.05, one-way ANOVA with *post hoc* Bonferroni^h^; Fig. 4*F*), which suggests that the rapid weight regain and the trend of reduced modified Irwin test score on day 4 observed in urethane-treated rats is not due to lower brain exposure to DFP in these rats. Together, these data suggest an early beneficial role of urethane to effectively terminate SE induced by OP-based compounds that allows rats to regain weight faster in the days following the initial insult.

### Hippocampal neurodegeneration is attenuated by urethane

**Figure 5.**
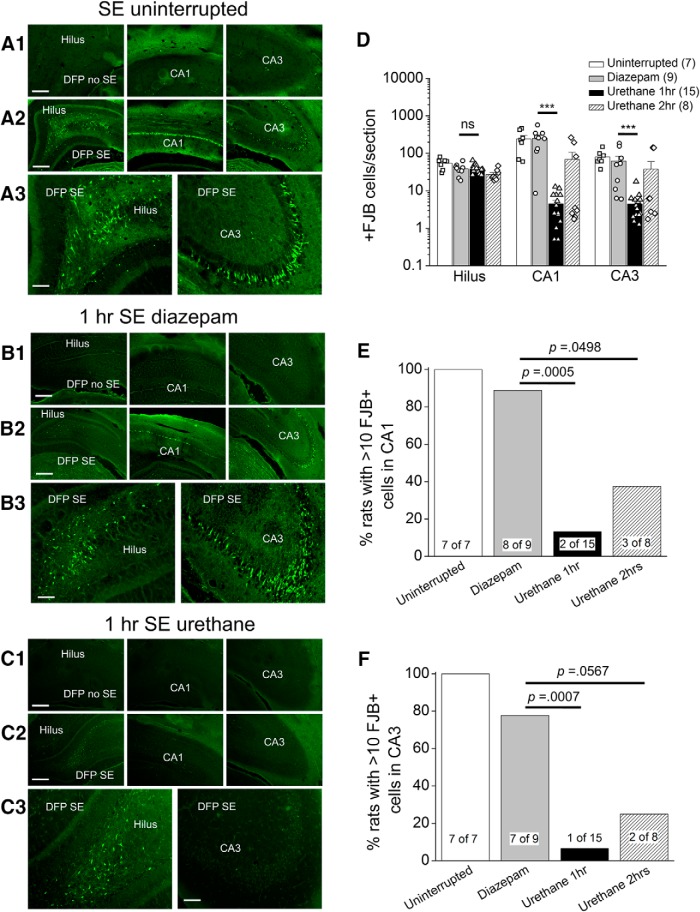
Hippocampal neuroprotection by urethane following DFP-induced SE. Representative images of FJB staining in hippocampal sections (40 µm) in the hilus, CA1 and CA3 regions 4 d after DFP-induced SE for rats that experienced uninterrupted SE (***A***), rats treated with diazepam after 1 h of SE (***B***), and rats injected with urethane after 1 h of SE (***C***). Each panel contains representative images of hippocampal sections taken from rats administered DFP that did not experience SE (DFP no SE). Images were taken with a 5× objective lens for the one and two series and a 20× objective lens for three. The images are representative of four dorsal hippocampal sections per rat. Scale bar = 300 μm for series 1 and 2, and 75 μm for series 3. ***D***, the average number of injured neurons per section 4 d after DFP-induced SE in three hippocampal regions (hilus, CA1, and CA3) of rats that experienced uninterrupted SE (white bar, open squares, *n* = 7 rats), rats treated with diazepam following 1 h of SE (gray bar, open circles, *n* = 9 rats), rats injected with urethane after 1 h of SE (black bar, open triangles, *n* = 15 rats), and rats injected with urethane after 2 h of SE (slashed bar, open diamonds, *n* = 8 rats; ***p < 0.001 in CA1 and CA3, the Mann–Whitney test was used to compare urethane to diazepam for rats that experienced 1 h of SE); ns = *p* > 0.05 by one-way ANOVA with *post hoc* Bonferroni. Data are the mean ± SEM. The symbols represent each individual rat within the group. The percentage of rats with 11 or more FluoroJade positive cells in the CA1 (***E***) and CA3 (***F***) regions of the hippocampus is shown for each treatment group (statistical significance is determined by *p* < 0.05, Fisher’s exact test comparing diazepam to urethane). The number of rats in each group is shown within the bars.

SE induced by DFP produces a characteristic pattern of neuron loss in the hippocampus of adult rats (Kadriu et al., 2011; Li et al., 2011, 2012; Rojas et al., 2015). To determine the extent of neuronal injury, FJB staining was performed on coronal hippocampal sections (40 μm) taken from the brains of rats 4 d following DFP-induced SE. Robust neurodegeneration in the CA1 region of the hippocampus was observed 4 d after uninterrupted SE (Fig. 5*A*,*D–F*), consistent with a recent study by Rojas et al. (2015). To determine whether pharmacological interruption of SE after 1 or 2 h reduces hippocampal neurodegeneration measured 4 d after DFP exposure, FJB-positive cells were counted in the hilus, CA1 and CA3 in rats administered either diazepam or urethane. In the CA1 region the total number of FJB-positive cells per section was significantly lower in rats administered urethane 1 h after DFP-induced SE compared to rats administered diazepam (254 ± 54 FJB-positive cells per hippocampal section for diazepam, *n* = 9 rats vs 5 ± 1 for urethane, *n* = 15 rats; *p* = 0.0001, Mann–Whitney test^i^; Fig. 5*B–D*). Reduced neurodegeneration was also observed in the CA3 region of urethane-treated rats after 1 h of SE (62 ± 22 FJB-positive cells per hippocampal section for diazepam, *n* = 9 rats vs 5 ± 1 for urethane, *n* = 15 rats; *p* = 0.0003, Mann–Whitney test^j^; Fig. 5*D*). A comparison of the number of rats with >10 FJB-positive cells per section in the CA1 region revealed significant hippocampal neuroprotection in rats injected with urethane (two out of 15 rats) following 1 h of DFP-induced SE compared to rats given diazepam (eight out of nine rats; *p* = 0.0005, Fisher’s exact test^k^; Fig. 5*E*). Similarly, the number of rats with >10 FJB-positive cells in CA3 was lower in urethane (one out of 15 rats) treated rats compared to diazepam (seven out of nine rats; *p* = 0.0007, Fisher’s exact test^l^; Fig. 5*F*). Although neurodegeneration was detected in the hilus of all rats using FJB, there was no difference in the number of FJB-positive cells per section of diazepam-treated rats compared to urethane-treated rats that experienced 1 h of SE (38 ± 4 cells per section for diazepam-treated rats, *n* = 9 vs 38 ± 3 for urethane-treated rats, *n* = 15; *p* > 0.05, Student’s *t* test^m^; Fig. 5*D*), likely because hilar cell death occurs rapidly after SE (Borges et al., 2003). Delayed administration of urethane (2 h after SE onset) resulted in a trend for neuroprotection in the CA1 (3 out of 8 rats) and CA3 (two out of eight rats), but statistical significance was not attained when compared to rats administered diazepam following 1 h of SE (*p* = 0.0498 for CA1 and *p* = 0.0567 for CA3, Fisher’s exact test^n^; Fig. 5*D–F*). Neurodegeneration was not observed in rats that were injected with DFP but did not enter into SE (Fig. 5*A–C*) or non-seizure control rats that were administered sterile water instead of DFP (data not shown). Together, these data indicate that early termination of DFP-induced SE by urethane exposure significantly reduces hippocampal neurodegeneration.

### Urethane blunts the expression of inflammatory mediators following DFP-induced SE

**Figure 6.**
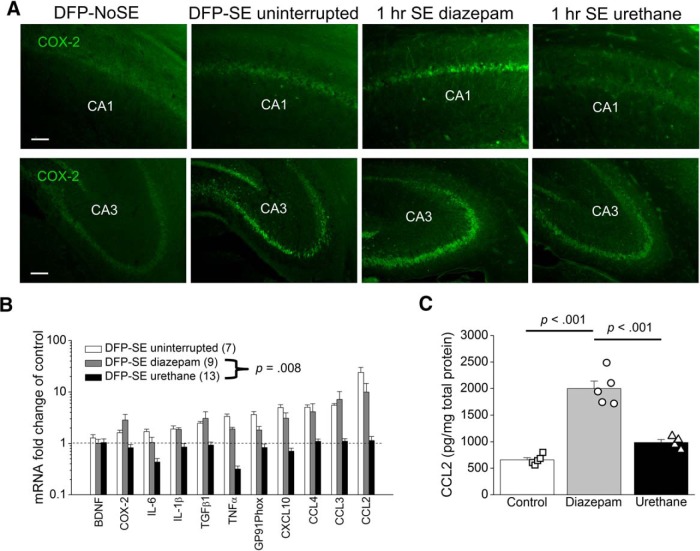
Induction of brain inflammatory mediators 4 d after DFP-induced SE in rats is attenuated by urethane. Fluorescence images (***A***) taken from the CA1 (top) and CA3 (bottom) regions in the hippocampus reveals basal expression of neuronal COX-2 in rats that did not experience SE (DFP-No SE). COX-2 expression is induced to the same degree in rats that experienced uninterrupted SE and rats administered diazepam 1 h following SE onset. Rats administered urethane 1 h following DFP-induced SE onset display lower induction of COX-2 in the CA1 and CA3 regions compared to diazepam. The images shown are a single representative of five hippocampal sections each from three rats in the groups. Scale bar = 100 μm. ***B***, Change in abundance of 11 inflammatory mediator mRNAs from the forebrain of rats 4 d after injection with water or DFP to induce SE. Following DFP-induced SE, the mRNA fold change for all 11 mediators as a group was significantly reduced by urethane compared to diazepam (*p* = 0.01, paired *t* test). Data are the mean ± SEM. ***C***, Changes in CCL2 protein in the brains of rats following DFP exposure measured 4 d after DFP-induced SE by ELISA. Data are the mean ± SEM. The symbols represent each individual rat within the group (*n* = 4–5 rats for each group; *p* < 0.001, one-way ANOVA with *post hoc* Bonferroni).

Upregulation of inflammatory genes is seen in rats 4 d after DFP-induced SE (Rojas et al., 2015). COX-2 induction is an important component of this inflammatory cascade (Serrano et al., 2011; Jiang et al., 2013; Rojas et al., 2015). In the current study rats administered DFP were euthanized 4 d after the onset of SE and one hemisphere was processed for IHC. Fluorescence IHC performed on coronal hippocampal sections (40 µm) revealed COX-2 upregulation in the hippocampus 4 d following DFP-induced SE compared to rats that received DFP but did not enter SE (Fig. 6*A*). The degree of COX-2 induction in hippocampal pyramidal neurons (CA1 and CA3) appeared to be reduced in rats injected with urethane 1 h after SE onset compared to rats that received diazepam and rats that experienced uninterrupted SE (Fig. 6*A*).

qRT-PCR was then conducted to measure expression in total brain tissue (minus cerebellum) of a selected panel of 11 inflammatory mediators (Table 2), a majority of which were previously shown to be upregulated in the rat brain following uninterrupted DFP induced SE (Rojas et al., 2015). Here, rats that experienced uninterrupted SE 4 d earlier showed increased brain mRNA levels in ten of the 11 inflammatory mediators, but not the three housekeeping genes (β-actin, GAPDH and HPRT1; Table 3), including 24-fold for CCL2, 6-fold for CCL3, 5-fold for CCL4, 5-fold for CXCL10, 3-fold for TNFα, and 2-fold for IL-1β (Fig. 6*B*). However, rats administered urethane 1 h after DFP-induced SE onset displayed a broadly blunted inflammatory response measured on day 4, whereas most inflammatory genes were upregulated in rats administered diazepam (Fig. 6*B*). The average change of all 11 inflammatory mediators combined was significantly lower in the urethane-treated rats, with the seizure-induced upregulation being abolished 4 d after DFP-induced SE (3.5 ± 0.8 average fold induction for diazepam, *n* = 9 rats vs 0.8 ± 0.1 average fold induction for urethane, *n* = 13 rats, *p* = 0.008, paired *t* test^o^; Fig. 6*B*). The level of CCL2 protein on day 4 in forebrain homogenates was significantly higher in rats administered diazepam (*n* = 5 rats) compared to rats administered urethane (*n* = 4 rats; 2000 ± 140 pg/mg total protein for diazepam vs 985 ± 57 pg/mg total protein for urethane, *p* < 0.001, one-way ANOVA with Bonferroni *post hoc* test^p^) 1 h after SE onset (Fig. 6*C*). Rats injected with urethane 1 h after SE displayed similar levels of CCL2 to non-seizure controls (660 ± 41 pg/mg total protein for controls, *n* = 5 rats) as measured on day 4 (Fig. 6*C*). These data show that exposure of rats to DFP and subsequent SE induces an inflammatory cascade involving COX-2 and CCL2; however, urethane but not diazepam administration after SE completely blunts the inflammatory response normally observed 4 d later.

### Urethane treatment reduces astrogliosis

**Figure 7.**
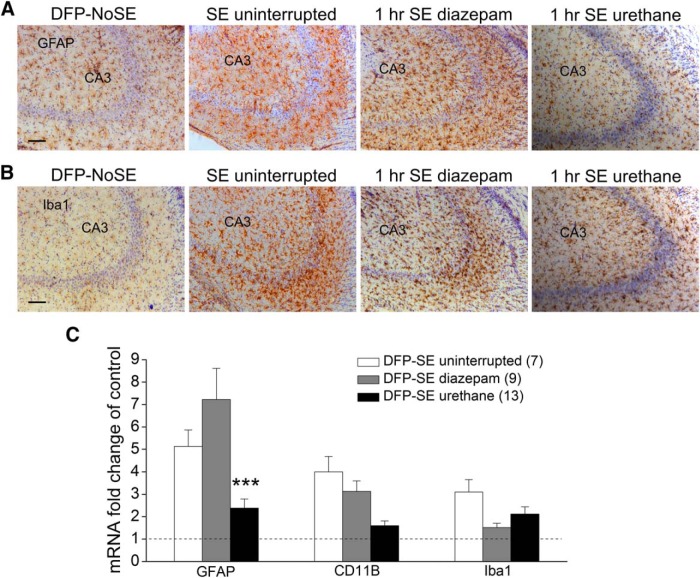
DFP-induced astrogliosis is reduced by urethane. Representative images (taken with a 10× objective lens) showing positive GFAP immunostaining (***A***) as an astrocyte marker and Iba1 immunofluorescence staining (***B***) as a microglial marker in the hippocampal CA3 region (DAB with a Nissl counterstain). Four days after DFP-induced SE, astrogliosis, and microgliosis were obvious in the sections obtained from rats as defined by the increased number of positively labeled cells in rats that experienced uninterrupted SE and rats administered diazepam 1 h after SE onset but not as much in sections taken from rats injected with urethane 1 h following SE onset or non-seizure controls. Scale bar, 100 μm. ***C***, Induction of GFAP, CD11B and Iba1 mRNA in the forebrain 4 d following DFP SE in rats that experienced uninterrupted SE (*n* = 7), rats administered diazepam 1 h after SE onset-treated (*n* = 9 rats) and urethane-treated (1 h SE) rats (*n* = 13 rats; ***p < 0.001, one-way ANOVA with *post hoc* Bonferroni).

In rats astrogliosis and microgliosis are prominent in the hippocampus following DFP-induced SE (Liu et al., 2012; Rojas et al., 2015; Flannery et al., 2016). Experiments were performed to measure astrogliosis and microgliosis in rats that received diazepam or urethane after DFP-SE 4 d earlier. Low levels of GFAP and Iba1 in the CA3 region of the hippocampus were observed in rats that were administered DFP but did not enter SE (Fig. 7*A*,*B*). Both GFAP and Iba1 were upregulated in the CA3 region of the hippocampus in rats administered diazepam 1 h after DFP-induced SE onset or rats that experienced uninterrupted SE 4 d earlier (Fig. 7*A*,*B*). However, the degree of GFAP and Iba1 induction in CA3 appeared to be reduced in rats injected with urethane 1 h after SE onset (Fig. 7*A*,*B*). qRT-PCR was used to quantify changes in mRNA of astrocytic GFAP, microglial CD11B and microglial Iba1 in rat brains after SE that can be indicative of the level of gliosis. Consistent with a recent study by Rojas et al. (2015) mRNA for all three glial markers were upregulated in rats that experienced uninterrupted SE 4 d earlier (Fig. 7*C*). However, rats administered urethane 1 h after SE displayed a reduced level of mRNA for GFAP compared to rats injected with diazepam [7.2 ± 1.4-fold over control (*n* = 9 rats) for diazepam vs 2.4 ± 0.4 (*n* = 13 rats) for urethane; *p* < 0.001, one-way ANOVA with *post hoc* Bonferroni^q^; Fig. 7*C*]. A strong trend for reduced CD11B mRNA was also observed in the brains of urethane-treated rats [3.1 ± 0.5 (*n* = 9 rats) for diazepam vs 1.6 ± 0.2 (*n* = 13 rats) for urethane], although statistical significance was not attained (*p* > 0.05, one-way ANOVA with *post hoc* Bonferroni^r^; Fig. 7*C*). The level of Iba1 mRNA, although increased in rats that experienced uninterrupted SE, was reduced in rats administered urethane or diazepam to the same degree [1.5 ± 0.2 (*n* = 9 rats) for diazepam vs 2.1 ± 0.3 (*n* = 13 rats) for urethane; Fig. 7*C*], suggesting that the effect of urethane may be more pronounced on astrocytes compared to microglia 4 d after DFP.

### Urethane and diazepam have similar effects on behavior in light/dark exploration and NOR

**Figure 8.**
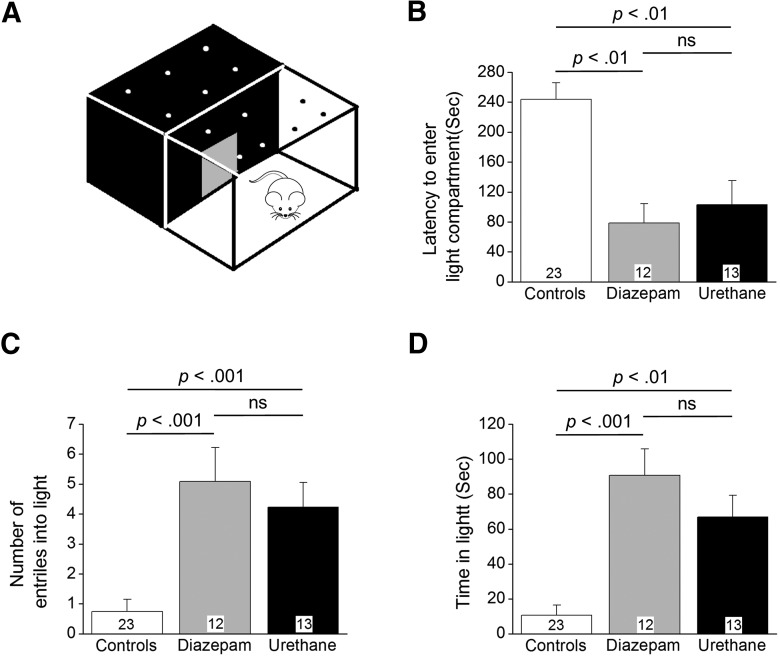
Urethane and diazepam have similar effects on exploration and anxiety behavior four to five weeks following SE. ***A***, Schematic of light/dark exploration apparatus. Latency to enter the light compartment (***B***), number of entries into the light compartment (***C***), and the time spent in the light compartment (***D***) are shown for the three groups of rats (controls, DFP-SE followed by diazepam, DFP-SE followed by urethane). The bars show the mean of the group and the number in the white box within the bar represents the total number of rats in each group. The error bars represent SEM; *p* < 0.01 and *p* < 0.001, Kruskal–Wallis test with Dunn’s *post hoc*; ns = *p* > 0.05 by Kruskal–Wallis test with Dunn’s *post hoc*. Grubb’s test did not identify any outliers.

Experiments were performed to determine whether early pharmacological interruption of SE alters long-term cognitive deficits in rats that endure 1 h of DFP-induced SE and survive. The initial behavioral analysis was conducted four weeks after DFP-induced SE in a light/dark preference apparatus (Fig. 8*A*). We chose four weeks as an appropriate time to investigate the behavior of the rats for the following reasons: (1) by week 4, all rats had recovered from the SE experience and surgery as assessed by weight regain and the modified Irwin score; and (2) by week 4, we had not observed generalized behavioral seizures in the rats administered diazepam or urethane. It was previously shown that DFP does not affect long-term anxiety behaviors in this test in rats that do not experience SE (Rojas et al., 2016). Rats that experienced SE and were administered diazepam behaved similarly as urethane-injected rats in the light/dark exploration task as shown in Figure 8, suggesting that termination or disruption of SE does not alter long-term anxiety behaviors. However, rats that endured SE spent significantly more time in the lit compartment of the light/dark box compared to control rats [91 ± 15 s for DFP-SE followed by diazepam (*n* = 12 rats), 67 ± 12 s DFP-SE followed by urethane (*n* = 13 rats) vs 11 ± 6 s for controls, *n* = 23 rats); *p* < 0.001 control vs diazepam; *p* < 0.01 control vs urethane by Kruskal–Wallis test with *post hoc* Dunn’s^s^; Fig. 8*D*]. It should be noted that all rats regardless of treatment had a preference for the dark as most of their time in the apparatus was spent in the dark.

**Figure 9.**
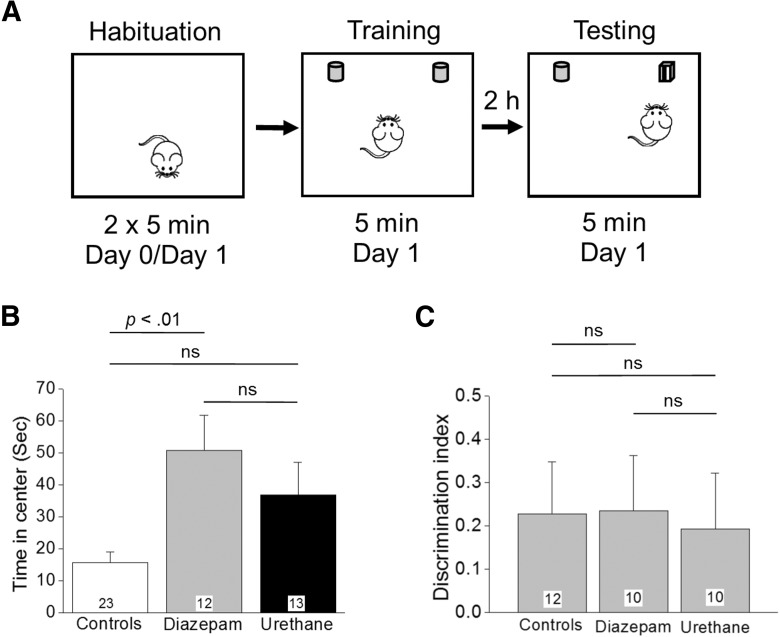
NOR memory in rats that experienced SE is unaffected by urethane. ***A***, Schematic of NOR testing consisting of three epochs conducted over 2 d. ***B***, Time spent in the center during the habituation is shown for the three groups tested; *p* < 0.01, by Kruskal–Wallis test with *post hoc* Dunn’s. ***C***, A DI was used as a measure of memory retention. The number in the white box within the bar represents the total number of rats in each group; ns = *p* > 0.05, one-way ANOVA with Bonferroni *post hoc*. Rats that spent <30% of the exploration time with either identical object did not familiarize during the training session and were deemed not fit to perform NOR testing. These rats were not included in the analysis of the DI reducing the total number of rats to that shown within the bars for each group.

To determine whether termination of DFP-induced SE in rats affects memory formation we performed NOR testing four to five weeks after the SE experience on rats that had received a single injection of diazepam or urethane 1 h after SE onset. On the day before NOR testing, all rats were subjected to a habituation trial in the NOR arena. This habituation trial was analyzed as an open field exploration test as the rats were allowed to freely move about the empty box and explore (Fig. 9*A*). Rats administered diazepam spent nearly the same amount of time in the center of the arena (an area defined as a 20 cm square in the very middle) as rats injected with urethane (51 ± 11 s in the center for diazepam, *n* = 12 rats vs 37 ± 10 s in the center for urethane, *n* = 13 rats; *p* > 0.05, by Kruskal–Wallis test with *post hoc* Dunn’s^t^; Fig. 9*B*). However, the control rats spent less time in the center of the arena (16 ± 3 s, *n* = 23 rats; Fig. 9*B*) compared to the rats that had experienced SE four to five weeks earlier. The open field data are consistent with the results from the light/dark exploration.

We then compared NOR performance among three experimental cohorts of rats to investigate whether interruption of SE rescues impaired memory following DFP-induced SE. The experimental cohorts were as follows: (1) non-seizure control rats, (2) DFP-SE rats that received diazepam, and (3) DFP-SE rats that received urethane. Rats from all three cohorts spent a similar percentage of time exploring both identical objects during the training phase. The percentage of time spent exploring the identical object on the left during familiarization was found to be 49 ± 4%, for controls (*n* = 12 rats), 47 ± 4%, for diazepam (*n* = 10 rats), and 52 ± 3% for urethane (*n* = 10 rats). The data from the NOR testing revealed that rats from all three groups spent the same time exploring the novel object compared to the familiar object with an average DI of 0.23 ± 0.1, *n* = 12 for control rats, 0.23 ± 0.1, *n* = 10 for diazepam injected rats, and 0.19 ± 0.1, *n* = 10 for urethane-injected rats (*p* > 0.05, one-way ANOVA with *post hoc* Bonferroni^u^; Fig. 9*C*), indicating that all of the rats remembered the familiar object from the object familiarization phase and thus had similar preference for the novel object. Together, these data indicate that under the given experimental conditions 1 h of DFP-induced SE was not sufficient to induce recognition memory deficits brought out in the NOR task regardless of posttreatment.

### Urethane reduces the occurrence of spontaneous recurrent seizures

**Figure 10.**
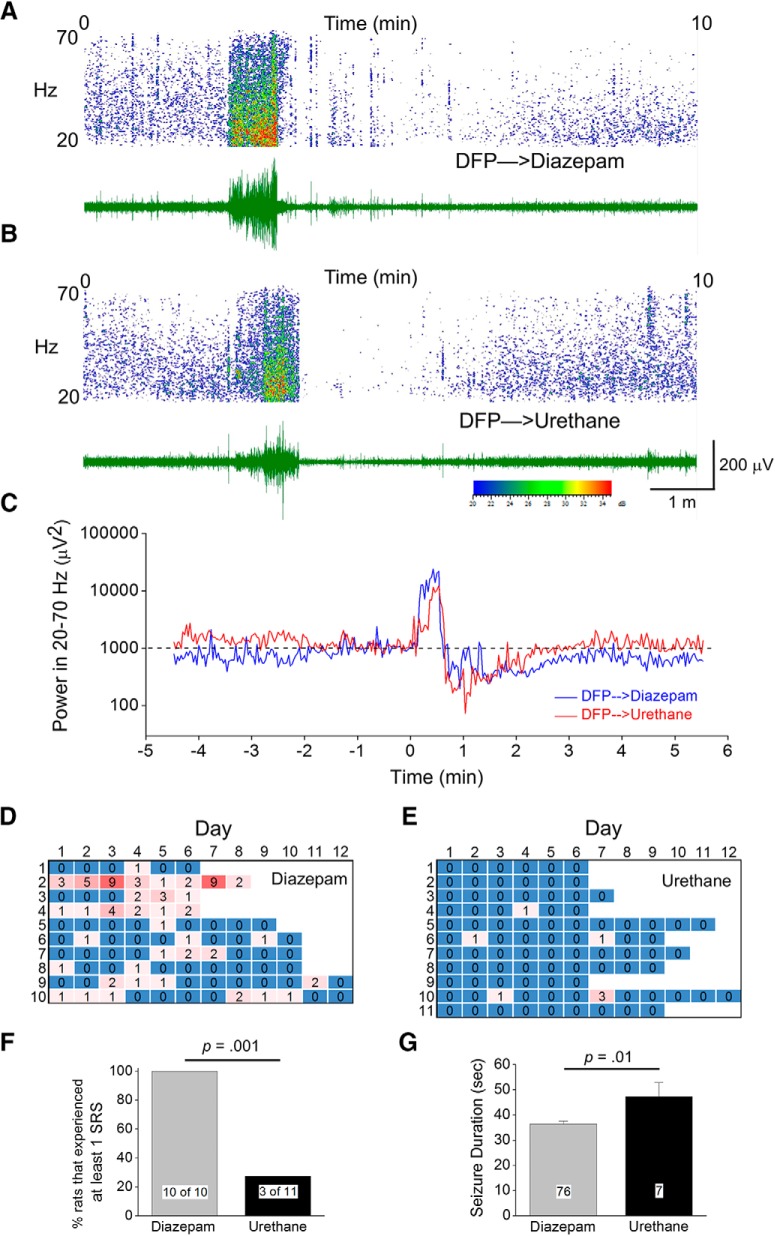
Urethane reduces the frequency of spontaneous recurrent seizures six to eight weeks following 1 h of DFP-induced SE. ***A***, Cortical EEG activity was recorded four to six weeks after SE induction by exposure to DFP. A representative 10**-**min EEG trace in green from the cortical recording of an adult rat that received diazepam 1 h after SE showed spontaneous rapid increased spike activity that lasted ∼40 s with a fast termination followed by a post-ictal depression. ***B***, A similar EEG trace in green from the cortical recording of a second adult male rat that received urethane 1 h after SE onset showing a similar seizure. Above each raw EEG trace in ***A***, ***B*** is a sonogram of the spike activity in the 20- to 70**-**Hz bandwidth obtained in Spike2. The colors of the sonogram indicate the spectral power density in decibels (dB, inset) at the indicated frequency. ***C***, Diagram showing the total EEG power in the 20- to 70**-**Hz bandwidth during the 10**-**min period that includes the SRS in ***A***, ***B*** using power analysis software written in Python. The dashed line indicates no spiking activity (baseline). Note that immediately after the spiking stops the baseline of both traces becomes quiet for ∼2 min representing the post-ictal depression. ***D***, ***E***, Heat maps showing the number of seizures detected per day from each rat. ***F***, A difference was detected between the two treatment groups [diazepam (*n* = 10) and urethane (*n* = 11)] in the percentage of rats that experienced at least one SRS during the 6–12 d of EEG recording; *p* = 0.001, Fisher’s exact test. ***G***, A difference was also detected between the average seizure duration for the two treatment groups [diazepam (*n* = 10) and urethane (*n* = 11)]. Error bars show SEM; *p* = 0.01, Student’s *t* test. The total number of seizures is shown within the bars for each group.

SE induced by nerve agents in animals leads to the development of spontaneous recurrent seizures (de Araujo Furtado et al., 2010, 2012). Adult male and female rats that experienced DFP-induced SE were administered diazepam or urethane 1 h after SE and allowed to recover. Two weeks after SE the rats were implanted with cortical electrodes and continuous 24-h cortical EEG recording for 6–12 d was performed two to three weeks later. All rats displayed normal cortical activity before and after ictal events as determined by the low amplitude and low power of the waveforms in the 20- to 70-Hz band (Fig. 10*A*,*B*). Typical SRS were defined by the appearance of a gradually-intensifying burst of large-amplitude (>3× the baseline) and high-frequency spikes that persisted for >20 s followed by a rapid quieting of electrical activity termed the “post-ictal depression.” SRS were found in both diazepam and urethane-injected rats (Fig. 10*A*,*B*). Using a script written in Python the power defined as the square of the spike amplitude in the 20- to 70-Hz band was obtained for seizures (Fig. 10*C*). Both diazepam and urethane-injected rats displayed multiple SRS (Fig. 10*D*,*E*). However, only 27% (three out of 11 rats) of the urethane-injected group experienced a SRS during the experiment compared to 100% (10 out of 10 rats) for diazepam (Fig. 10*F*; *p* = 0.001 by Fisher’s exact test^v^). Moreover, only seven seizures were detected for the three urethane-injected rats, whereas 76 total seizures were counted in 10 diazepam-treated rats (Fig. 10*D*,*E*). The duration of spontaneous seizures, defined as the period in which spikes persisted from the initial spike to the time of the last detectable spike just before the post-ictal depression, was significantly lower in the diazepam injected group compared to urethane [36 ± 1 s for diazepam (*n* = 76 seizures) vs 47 ± 6 s for urethane (*n* = 7 seizures); *p* = 0.01, Student’s *t* test^w^; Fig. 10*G*]. However, we believe that this difference may be attributed to the fact that 2 of the 7 SRS detected in the urethane group were abnormally long (55 and 72 s), whereas the other five seizures were very similar in duration to the average SRS in the diazepam group. Indeed, with a statistical power of only 55% (Table 4), this result is likely to be a false positive. Together, these data demonstrate that rats treated post-SE with urethane experienced a lower incidence and frequency of SRS following DFP-induced SE, compared with diazepam-treated rats.

### No evidence of lung tumors was detected following a single subanesthetic dose of urethane

**Figure 11.**
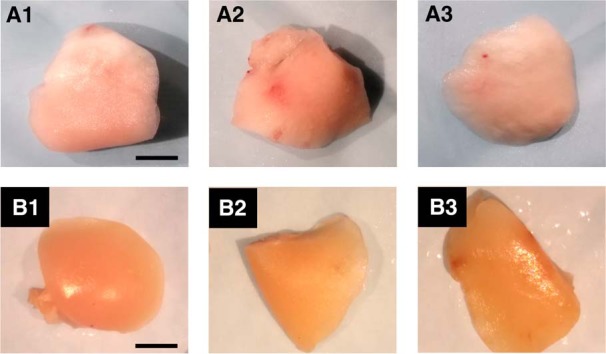
A single subanesthetic dose of urethane does not produce lung tumors in rats. Representative photographs (***A***) of individual perfused lung lobes obtained from naïve control rats. A single lobe from three of eight rats is shown (scale bar, 0.7 cm) and **(*B***) of individual perfused rat lung lobes seven months following a single dose of urethane (0.8 g/kg, s.c.). A single lobe from three of five treated rats is shown. None of the lobes displays evidence of lung adenomas as the surfaces were smooth and void of any cancerous outgrowths similar to the lobes of the naïve controls. Scale bar = 0.7 cm.

Exposure to high doses of urethane has been shown to induce lung tumors (adenomas) in rats (Guyer and Claus, 1947; Jaffe and Jaffe, 1947; Rosin, 1949; Tannenbaum et al., 1962; Field and Lang, 1988). The use of urethane to induce lung tumors is a simple and commonly used animal model of lung cancer. Experiments were performed to determine whether a single subanesthetic dose of urethane led to the appearance of adenoma lung tumors in adult rats. Five adult male Sprague Dawley rats were administered a single subanesthetic dose (0.8 g/kg) of urethane and observed frequently (at least once a week) for seven months. During the seven-month period, the rats administered urethane displayed normal breathing and behavior as determined visually. After seven months the rats were euthanized and the lungs were collected to identify possible adenomas. The lungs obtained from urethane-injected rats were compared to lungs obtained from naïve controls. Visual gross observation of individual lung lobes revealed no evidence of adenomas after a single subanesthetic dose of urethane in all five rats. Individual lung lobes from three out of eight total naïve control rats in Figure 11*A* were compared to individual lung lobes obtained from three out of five urethane-injected rats in Figure 11*B*. None of the lungs obtained from the rats showed evidence of adenomas as the surface of the lungs was very smooth and free of adenoma nodules or cancerous outgrowths. On the other hand, adenoma modules are present and obvious as early as 8–10 weeks on the surface of lung lobes obtained from rats that received repeated intraperitoneal injections of urethane (Guyer and Claus, 1947).

## Discussion

An optimized model of DFP exposure producing SE in adult male rats was used to investigate the early and late functional outcomes of interrupting SE by diazepam and urethane. In the rat DFP model of SE we found that diazepam suppressed SE initially, but inevitably seizure activity with high power in the 20- to 70-Hz band returned within 6–10 h after diazepam administration. On the other hand, a single subanesthetic dose of urethane administered 1 h after DFP-induced SE resulted in sustained suppression of seizure activity over the 24–48 h recording. The return of high-power seizure activity was abolished by urethane even if administration were delayed to 2 h after SE onset. The benefits of early termination of electrographic SE with urethane compared to diazepam was multifaceted, including accelerated recovery from weight loss, less hippocampal neurodegeneration, reduced neuroinflammation and less astrogliosis by day 4 after DFP exposure. Interestingly, although rats administered diazepam displayed a return of high-power seizure activity following SE they, like rats administered urethane, typically survived the next 4 d. Administration of diazepam during SE has been shown to improve survival in other models of SE (Mello et al., 1993; Apland et al., 2014; Trandafir et al., 2015). On the other hand, rats that experienced uninterrupted SE displayed a high mortality. Therefore, terminating SE with either urethane or diazepam promotes survival. The return of SE and not the initial bout of SE in this rat model of DFP-induced SE appears to underpin the intermediate and late consequences of SE. Brandt et al. (2015) demonstrated that a longer duration of initial SE causes more intense sequelae; our work extends this to show that overnight return of high-intensity seizures causes most of the pathologic consequences of SE. We propose that this second, delayed, period of seizures occurring during a metabolically-vulnerable state caused by the initial bout of SE is responsible for neurodegeneration and accompanying inflammation.

Spontaneous recurrent seizures were observed six to eight weeks after DFP-induced SE in rats treated with either diazepam or urethane. A single administration of urethane during SE reduced the proportion of rats that displayed spontaneous recurrent seizures by 73% and the frequency of SRS by 90% compared to diazepam-treated rats. Since we did not perform EEG recordings on the rats continuously from the day of SE we were unable to determine whether the onset of SRS was delayed by urethane treatment. It should be noted that two rats administered diazepam after 1 h of SE four weeks earlier re-entered SE during SRS recordings (data not shown). Both rats died as a result of the complications that arose following this second bout of SE. None of the urethane-injected rats experienced a return of SE during the monitoring of SRS. Together, we have shown that a single subanesthetic dose of urethane administered 1 h after DFP-induced SE produces long-term beneficial consequences.

A common neuropathology associated with DFP-induced SE is neurodegeneration in the hippocampus (Kadriu et al., 2011; Li et al., 2011, 2012; Liu et al., 2012; Rojas et al., 2015). The variation observed in neurodegeneration in the pyramidal cell layers of the hippocampus (CA1 and CA3; Fig. 5*D*) was large. Similarly, the variation in the duration and intensity of the high-power seizure activity seen in the activity return phase of the EEG was also large. However, direct correlation could not be determined as the neurodegeneration and EEG data were obtained from different cohorts of rats. Nevertheless, it is plausible that the overnight reinstatement of high-power seizure activity may contribute to delayed hippocampal pyramidal cell neurodegeneration in the rat DFP model. Rats that experienced SE terminated by urethane also displayed no evidence of an inflammatory burst in the brain on day 4 as determined by RT-PCR of 11 inflammatory mediators. On the other hand the inflammatory burst observed in diazepam injected rats was similar to that of rats that experienced uninterrupted SE. Gliosis was also prominent in the forebrain 4 d after DFP-induced SE that was uninterrupted, consistent with previous studies (Liu et al., 2012; Rojas et al., 2015; Flannery et al., 2016). Administration of urethane but not diazepam after 1 h of SE greatly diminished both astrogliosis and microglial activation in the brain (Fig. 7), supporting the idea that termination of DFP-induced SE and subsequent attenuation of the return of high-power seizure activity results in a lessening of gliosis. Importantly the initial SE experience was similar for urethane and diazepam administered rats.

The dose of urethane used in the current study (0.8 g/kg) is subanesthetic; however, it produces a long lasting sedation that wears off the next day as rats began to move about the cage to obtain food and water. Although the rats administered diazepam recovered from sedation and were able to move about the cage earlier than rats given urethane, they did not regain weight as fast as the urethane administered rats (Fig. 4*C*). Recently, it was shown that prolonged anesthesia with isoflurane prevents acquired epilepsy in two rat models of temporal lobe epilepsy (systemic administration of paraoxon and intrahippocampal kainite; Bar-Klein et al., 2016). In the same study, isoflurane exposure reduced neurodegeneration in rats that experienced SE induced by paraoxon exposure and reduced neuroinflammation in the intrahippocampal kainate model (Bar-Klein et al., 2016). However, the long-term sedation and quieting of the EEG in the current study was clearly a result of the urethane exposure and not isoflurane. Unlike isoflurane, an anesthetic dose of urethane produces a long-lasting deep level of anesthesia without affecting the autonomic and cardiovascular systems (Soma, 1983; Maggi and Meli, 1986). The molecular mechanisms underpinning the anesthetic effects of urethane involve inhibition of excitatory neurotransmitter receptors and potentiation of inhibitory transmitter receptors (Hara and Harris, 2002). By reducing excitation via glutamate receptor antagonism and enhancing inhibition via potentiation of GABA receptors, urethane-like inhalational anesthetics may reduce excitatory drive on neuronal firing and networks. This action of urethane appears to be model independent as urethane reduces epileptiform activity in rats that experience SE induced by DFP in the current study and blocks the development of amygdala kindled seizures in rodents (Cain et al., 1989, 1992) as well as spinal seizures evoked by sudden cooling in a toad isolated spinal cord preparation (Piña-Crespo and Daló, 1992). Felbamate, which is structurally and functionally similar to urethane, is a very effective anticonvulsant in partial epilepsies. However, felbamate is associated with a risk of aplastic anemia and liver failure in humans limiting its use. Benzodiazepines, unlike urethane or felbamate, act by enhancing the effect of the neurotransmitter GABA on GABA_A_ receptors. If given early (within 30 min of SE induction), benzodiazepines can be very effective at terminating SE. However, diazepam when administered 1 or 2 h after soman exposure was shown to be ineffective at reducing the total duration of SE within a 24-h period (Apland et al., 2014), consistent with the current study in which SE was induced by the soman surrogate, DFP. In the same study, a glutamate receptor antagonist was more effective at terminating the return of seizures compared to diazepam, which is also consistent with the results shown here.

Rodents that survive an OP agent exposure also exhibit long-term cognitive deficits (Tilson et al., 1990; Raveh et al., 2003; Gilat et al., 2005; Joosen et al., 2009; Tattersall, 2009; Chen, 2012; Rojas et al., 2016) that can be associated with early neurodegeneration and the neuronal plasticity that occurs in the brain after SE (McDonough et al., 1987; Filliat et al., 1999; Myhrer et al., 2005; Joosen et al., 2009; Chen, 2012). Whether early termination of SE and the reduced neurodegeneration observed following urethane administration compared to diazepam also ameliorates the long-term cognitive deficits after DFP-induced SE was investigated in the current study. Rats exposed to DFP displayed reduced anxiety behavior regardless of whether they received urethane or diazepam. Exposure of rats to an SE-producing dose of soman was reported to increase anxiety behavior (Apland et al., 2014; Prager et al., 2014, 2015), whereas exposure to sub-SE doses of other OP agents showed a trend toward decreased anxiety behaviors (Schulz et al., 1990; Wright et al., 2010) or had no effect (Nieminen et al., 1991; Valvassori et al., 2007). Furthermore, reduced anxiety behavior was also observed in rats that experienced uninterrupted SE induced by DFP (Rojas et al., 2016). Thus, the net effect of OP agents on anxiety-related behaviors could depend on the agent, its dose and the duration of SE. Nevertheless, both diazepam and urethane administration failed to reverse the reduced anxiety behavior four to five weeks after SE in either of the two assays (light/dark preference testing and habituation of NOR), suggesting that 1 h of DFP-induced SE is sufficient to produce long-term reduced anxiety behavior in rats.

Rats that endure uninterrupted SE induced by DFP display an inability to discriminate between a novel and familiar object (Rojas et al., 2016), in contrast to rats in which SE had been interrupted with diazepam or terminated with urethane after 1 h (Fig. 9). This difference in the ability to remember a familiar object in the two studies may be due to the timing of the testing or whether SE was interrupted. In the earlier study the NOR task was performed 6–12 weeks after uninterrupted SE (Rojas et al., 2016) whereas in the current study NOR was performed four to five weeks after SE that was terminated pharmacologically. The longer latency from SE and the duration of the initial insult could account for the development of cognitive deficits such as memory formation. In the future it will be important to determine the time-dependent induction of cognitive deficits following SE induced by organophosphate based agents.

A small cohort of female rats was included in this study to determine whether females and males differ in the development of long-term neuropathologies or epilepsy after DFP-induced SE. We detected no difference in the behavior of female rats compared to males in the behavioral assays regardless of whether they received urethane or diazepam. There was also no difference in the number of SRS between male and females. However, only a small number of female rats were included in these experiments preventing the use of statistical analysis. For example, only one of four females and two of seven males in the urethane group experienced SRS. In the future, larger groups of males and females will be necessary to determine whether there are sex related differences in this rat organophosphate model of SE and epilepsy.

## Conclusion

Exposure to high levels of DFP causes SE, which if left untreated results in neuropathologies and the inevitable development of epilepsy in surviving rats. The general anesthetic, urethane, effectively terminated electrographic SE, reduced neuroinflammation, neurodegeneration and astrogliosis, accelerated weight regain within 4 d after DFP exposure, and reduced the incidence and frequency of spontaneous recurrent seizures, all when compared to diazepam. However, the anxiety-related comorbidities were not rescued by urethane. We conclude that administration of urethane following OP agent-based SE has a number of beneficial consequences. We have shown that preventing the overnight return of the high-power seizure activity following SE reduced neurodegeneration in the hippocampus and brain inflammation following urethane administration, which resulted in its beneficial effects compared to diazepam. Together, these studies give insight into therapeutic modalities for the treatment of SE. The results of this study suggest a thorough review of previous work relating SE to the development of epilepsy to ensure that SE was indeed abolished pharmacologically without overnight return of high-power seizure activity. In the future it will be important to determine whether early urethane treatment also ameliorates the consequences of SE by other OP-based agents.

## Synthesis

Reviewing Editor: Tallie Z. Baram, University of California-Irvine Decisions are customarily a result of the Reviewing Editor and the peer reviewers coming together and discussing their recommendations until a consensus is reached. When revisions are invited, a fact-based synthesis statement explaining their decision and outlining what is needed to prepare a revision will be listed below. The following reviewer(s) agreed to reveal their identity: Heidi Grabenstatter

Both reviewers appreciated the comprehensive and thorough nature of the work, and noted its significance. Reviewer 2 pointed out design elements that need to be considered when assessing whether urethane truly modifies disease course or simply reduces the severity of the inciting insult. As the implications are significantly different, the statements in the paper require thought and modification.

Additional reviewer comments merit the authors' attention as they improve this generally excellent paper in the revision:

Reviewer 1:

1. For analysis of SRS, the timing of implantation and recordings is unclear. Please clarify the experimental time-line for this study.

2. Why were two doses of DFP and atropine given? WHich experiments do the different doses correspond with?

3. In the results section describing the NOR task, the last sentence states “... these data indicate that neither diazepam nor urethane treatment after 1 hour of DFP-induced SE improves the long-term ability of rats to form new memories.” However, since control and DFP groups were not significantly different, the more appropriate interpretation would be: under the given experimental conditions, 1 hour of DFP-induced SE was not sufficient to induce deficits in recognition memory, regardless of post-treatment.

4. Why was diazepam chosen for this study and not midazolam, which is approved for OP poisoning?

Reviewer 2:

Methods: The authors don't explain why the 20-70 Hz bandwidth range was chosen for SE analysis, nor do they cite the paper that established the method. This range is the optimum range to isolate field EPSPs (i.e., the high β-, low γ-band) and minimizes any recorded movement artifact, which EEG acquisition during SE is particularly prone to. Readers who may not be in vivo electrophysiologists need an explanation for the removal of both high- and low- frequency bands in the analysis.

The data that was also filtered between 1-100 Hz was presumably used for the spike detection analysis in the ‘chronic’ analysis for seizure frequency. Please clarify in method section.

Are the uninterrupted SE rats administered a vehicle-control injection (e.g., saline)?

Are the sections serially cut and stained (e.g., every 6th section ) through the length of the hippocampus for histopathology analyses? Can the authors explain where the means that are presented for cell counts come from? How many sections from how many animals?

Results: Urethane reduces the severity of SE, thus, it would follow that (1) fewer animals would develop epilepsy, (2) less hippocampal neurodegeneration and microglial activation would be observed, and (3) functional recovery would be improved. However, this is not disease modifying. If a standard injury was given and a potential therapy delivered then disease modifying or antiepileptic potential can be discussed. The authors do not monitor the progression of epileptogenesis or the pathologies inherent to chronic epilepsy, thus, they cannot speak to the potential anti-epileptogenic or disease modifying effect. The only histopathology was quantified at 4 days post SE (i.e., acute not chronic). Chronic 24/7 EEG recordings were conducted 4-5 weeks after DFP administration and were only acquired for 6-12 days. This duration of recording may not have fully captured the total percentage of animals that are spontaneously epileptic. Has the long-term natural history of the DFP model been electrophysiologically characterized in vivo? When do the animals typically develop SRS and at what rate? Some background on this may help the reader and give some perspective to the percentages that are presented for the time points chosen.

Reduction of the SE-induced alterations in histopathology, mRNA, seizure development, and functional recovery do not, necessarily, indicate a disease modifying effect. Changes in these measures are reflective of a less severe injury. In other words, all of these indices are directly correlated with duration of SE and are not necessarily related to urethane treatment. Duration of SE and subsequent results has previously been described (Brandt et al., 2015 Neurobio Dis).

These results are important especially related to the return of seizures and the reduction of power on what might be termed, ‘refractory SE.’ Very few labs focus on the entire length of SE following a neurotoxic insult and these authors have done a fine job of analyzing not only the first bout of recurrent seizures, but the return of seizures, demonstrating that urethane exerts its affects during this later stage of SE. Is this transient effect responsible for the observed changes in mRNA expression, histopathology, seizure expression, and functional recovery? That is very meaningful to the field, in general. It will be particularly interesting to see whether changes in early measures related to the presence of a second bout of seizures may be predictive of the development of spontaneous seizures.

The authors should be commended for investigating the potential side effects of urethane use via analysis of lung tumor development.

## Author Response

Response to reviewers

Reviewer 1:

1. For analysis of SRS, the timing of implantation and recordings is unclear. Please clarify the experimental time-line for this study.

We have included sentences in the methods section to clarify the experimental timeline for the SRS experiments. Page 17, second paragraph, lines 1-5

2. Why were two doses of DFP and atropine given? WHich experiments do the different doses correspond with?

Early in the study we injected normal adult male rats with DFP intraperitoneally at 9.5 mg/kg according to the protocol published in two previous studies (Rojas et al., 2015, 2016). A review of the DFP literature demonstrated that DFP injected at a lower dose (5 mg/kg) subcutaneously also results in prolonged SE in rats. We therefore, compared the two doses and two different routes of administration and found that the characteristics of SE (i.e. time to SE onset, duration of SE, 24 hr Irwin score, weight loss, mortality and 4 day pathology) did not differ. Due to the fact that subcutaneous injections reduce the chance of injecting DFP into a major organ that can confound the interpretation of the data we decided that injecting DFP subcutaneously at 5 mg/kg would be a better option. Most of the rats in the study (all diazepam and urethane administered) were administered DFP at 5 mg/kg subcutaneously. Only a small cohort of normal adult male rats (from the uninterrupted group) were administered DFP at 9.5 mg/kg intraperitoneally. These rats were sacrificed on day 4 and the neuropathology (neuroinflammation, neurodegeneration and gliosis) was not different from rats that received DFP at 5 mg/kg subcutaneously (also in the uninterrupted group). We have included sentences in the methods section to clarify this. Page 7, lines 15-20.

We also made a switch from methylatropine nitrate to ethylatropine bromide early in the study. These muscarinic receptor antagonists, neither of which appreciably crosses the blood-brain barrier, were administered to rats prior to DFP to reduce peripheral OP toxicity and increase survival of the rats following DFP exposure without altering the development of seizures. The availability of methylatropine nitrate was reduced commercially and the commercial price increased significantly. With the expertise of medicinal chemists in our laboratory we synthesized ethylatropine bromide which was inexpensive, environmentally friendly and simple to make. More importantly we found ethylatropine bromide to be a very effective peripherally restricted muscarinic antagonist in pilocarpine induced seizures (Rojas et al., 2017). Similarly it was found to be very effective in our DFP model of SE and thus we began using ethylatropine bromide synthesized in our lab in place of methylatropine nitrate.

3. In the results section describing the NOR task, the last sentence states “... these data indicate that neither diazepam nor urethane treatment after 1 hour of DFP-induced SE improves the long-term ability of rats to form new memories.” However, since control and DFP groups were not significantly different, the more appropriate interpretation would be: under the given experimental conditions, 1 hour of DFP-induced SE was not sufficient to induce deficits in recognition memory, regardless of post-treatment.

The sentences have been changed. Page 28, paragraph 2, lines 14-16

4. Why was diazepam chosen for this study and not midazolam, which is approved for OP poisoning?

We chose diazepam as it is still currently approved by the US Food and Drug Administration as a first line treatment drug for organophosphorus nerve agent poisoning and it is still part of the CHEMPACK as an anticonvulsant antidote for nerve agent intoxication. Also, there is a greater wealth of literature on the efficacy of diazepam to terminate SE induced by chemical convulsants compared to midazolam. Page 4, paragraph 1, lines 15-17

Reviewer 2:

Methods: The authors don't explain why the 20-70 Hz bandwidth range was chosen for SE analysis, nor do they cite the paper that established the method. This range is the optimum range to isolate field EPSPs (i.e., the high β-, low γ-band) and minimizes any recorded movement artifact, which EEG acquisition during SE is particularly prone to. Readers who may not be in vivo electrophysiologists need an explanation for the removal of bothx high- and low- frequency bands in the analysis.

The data that was also filtered between 1-100 Hz was presumably used for the spike detection analysis in the ‘chronic’ analysis for seizure frequency. Please clarify in method section.

We have added a sentence in the methods section explaining why the 20-70 Hz bandwidth range was chosen for SE analysis. We have also cited the relevant paper that established the method. Page 6, paragraph 1, lines 9-12

Are the uninterrupted SE rats administered a vehicle-control injection (e.g., saline)?

The uninterrupted rats were not administered a vehicle-control injection at the 60 minutes time point to ensure that SE was indeed uninterrupted. Page 8, paragraph 1, lines 4-5

Are the sections serially cut and stained (e.g., every 6th section) through the length of the hippocampus for histopathology analyses? Can the authors explain where the means that are presented for cell counts come from? How many sections from how many animals?

We clarified this in the revised manuscript. Every 12th hippocampal section from each rat brain was stained with FluoroJade B to label degenerating cells according to the manufacturer protocol as described by Schmued et al. (1997). Following FluoroJade B labeling, images of three hippocampal areas (i.e., hilus, CA1, CA3) were taken with a 5x objective lens using the AxioVision AC 4.1 software (Zeiss) from each of 4 sections in each rat, from the dorsal hippocampus between bregma -2.56 and -4.16 mm (Paxinos & Watson, 1986). The number of bright FJB positive cells in each hippocampal area was counted by an observer blinded to the treatment of the animals and the experimental conditions. The cell numbers were recorded and expressed as the mean and standard error of the mean number of positive FluoroJade B (+FJB) cells/section for each hippocampal area in each rat. FluoroJade B stained sections were quantified for 9 rats that received diazepam one hour post SE, 7 rats that experienced uninterrupted SE, 15 rats that received urethane one hour after SE and 8 rats that received urethane two hours post SE.

Page 11, paragraph 1, lines 10-12; Page 12 lines 1-10.

Results: Urethane reduces the severity of SE, thus, it would follow that (1) fewer animals would develop epilepsy, (2) less hippocampal neurodegeneration and microglial activation would be observed, and (3) functional recovery would be improved. However, this is not disease modifying. If a standard injury was given and a potential therapy delivered then disease modifying or antiepileptic potential can be discussed. The authors do not monitor the progression of epileptogenesis or the pathologies inherent to chronic epilepsy, thus, they cannot speak to the potential anti-epileptogenic or disease modifying effect. The only histopathology was quantified at 4 days post SE (i.e., acute not chronic). Chronic 24/7 EEG recordings were conducted 4-5 weeks after DFP administration and were only acquired for 6-12 days. This duration of recording may not have fully captured the total percentage of animals that are spontaneously epileptic. Has the long-term natural history of the DFP model been electrophysiologically characterized in vivo? When do the animals typically develop SRS and at what rate? Some background on this may help the reader and give some perspective to the percentages that are presented for the time points chosen.

The long-term consequences of DFP-induced SE in rodents have not been electrophysiologically characterized. Also, it has not been established as to when the animals typically develop spontaneous recurrent seizures and at what rate following DFP exposure. Although we have shown in the current study that fewer rats exhibited seizures and observed fewer seizures per epileptic rat following urethane administration post DFP-induced SE compared to diazepam injected rats, we did not record EEG continuously from the day of SE onward to investigate epileptogenesis. The reviewer is correct that the ability of urethane to terminate SE and block the development of refractory SE may lead to a lesser insult and account for the long term changes in spontaneous recurrent seizures. Therefore, we have modified the paper and removed statements suggesting a disease modifying effect of urethane. We now emphasize the ability of urethane to terminate SE following exposure to an OP based agent. This study suggests that the return of SE and not the initial bout of SE in this rat model of DFP-induced SE underscores the early, intermediate and late consequences of SE. Also, the results of this study suggest that in the future there should be a thorough review of previous work relating SE to the development of epilepsy to ensure that SE was indeed abolished pharmacologically with no return of high power overnight seizure activity following SE. The abstract, results and discussion sections have been modified accordingly.

Reduction of the SE-induced alterations in histopathology, mRNA, seizure development, and functional recovery do not, necessarily, indicate a disease modifying effect. Changes in these measures are reflective of a less severe injury. In other words, all of these indices are directly correlated with duration of SE and are not necessarily related to urethane treatment. Duration of SE and subsequent results has previously been described (Brandt et al., 2015 Neurobio Dis).

These results are important especially related to the return of seizures and the reduction of power on what might be termed, ‘refractory SE.’ Very few labs focus on the entire length of SE following a neurotoxic insult and these authors have done a fine job of analyzing not only the first bout of recurrent seizures, but the return of seizures, demonstrating that urethane exerts its affects during this later stage of SE. Is this transient effect responsible for the observed changes in mRNA expression, histopathology, seizure expression, and functional recovery? That is very meaningful to the field, in general. It will be particularly interesting to see whether changes in early measures related to the presence of a second bout of seizures may be predictive of the development of spontaneous seizures.

The authors should be commended for investigating the potential side effects of urethane use via analysis of lung tumor development.

We completely agree with the reviewer's conclusion that the overnight return of SE-like seizure activity, rather than the initial bout of SE, is responsible for most of the sequelae. This finding extends anecdotal reports by many, and the systematic study of Brandt et al (2015), that the duration of SE influences the intensity of its consequences.
